# Metabolic competition between host and pathogen dictates inflammasome responses to fungal infection

**DOI:** 10.1371/journal.ppat.1008695

**Published:** 2020-08-04

**Authors:** Timothy M. Tucey, Jiyoti Verma, Françios A. B. Olivier, Tricia L. Lo, Avril A. B. Robertson, Thomas Naderer, Ana Traven

**Affiliations:** 1 Infection and Immunity Program and the Department of Biochemistry and Molecular Biology, Biomedicine Discovery Institute, Monash University, Clayton, Victoria, Australia; 2 School of Chemistry and Molecular Biosciences, University of Queensland, Brisbane, Australia; Memorial Sloan-Kettering Cancer Center, UNITED STATES

## Abstract

The NLRP3 inflammasome has emerged as a central immune regulator that senses virulence factors expressed by microbial pathogens for triggering inflammation. Inflammation can be harmful and therefore this response must be tightly controlled. The mechanisms by which immune cells, such as macrophages, discriminate benign from pathogenic microbes to control the NLRP3 inflammasome remain poorly defined. Here we used live cell imaging coupled with a compendium of diverse clinical isolates to define how macrophages respond and activate NLRP3 when faced with the human yeast commensal and pathogen *Candida albicans*. We show that metabolic competition by *C*. *albicans*, rather than virulence traits such as hyphal formation, activates NLRP3 in macrophages. Inflammasome activation is triggered by glucose starvation in macrophages, which occurs when fungal load increases sufficiently to outcompete macrophages for glucose. Consistently, reducing *Candida*’s ability to compete for glucose and increasing glucose availability for macrophages tames inflammatory responses. We define the mechanistic requirements for glucose starvation-dependent inflammasome activation by *Candida* and show that it leads to inflammatory cytokine production, but it does not trigger pyroptotic macrophage death. Pyroptosis occurs only with some *Candida* isolates and only under specific experimental conditions, whereas inflammasome activation by glucose starvation is broadly relevant. In conclusion, macrophages use their metabolic status, specifically glucose metabolism, to sense fungal metabolic activity and activate NLRP3 when microbial load increases. Therefore, a major consequence of *Candida*-induced glucose starvation in macrophages is activation of inflammatory responses, with implications for understanding how metabolism modulates inflammation in fungal infections.

## Author summary

Activation of the immune regulator NLRP3 inflammasome by microbial pathogens has been shown to play both protective and destructive roles in infection, underscoring the importance of tight control over NLRP3-driven inflammation to ensure host health. A key microbe recognised by NLRP3 is the human yeast commensal and pathogen *Candida albicans*, which is responsible for mucosal and invasive infections. We demonstrate that innate immune cells sense their metabolic status to trigger NLRP3 activation only when microbial numbers have reached dangerous levels. This regulation is a consequence of metabolic competition between *C*. *albicans* and macrophages for an essential nutrient–glucose. The NLRP3 inflammasome is activated when increased fungal load in the infection microenvironment drives down glucose levels, thereby causing glucose starvation in macrophages. Restoring glucose homeostasis in macrophages reduced NLRP3 activation and production of the proinflammatory cytokine IL-1β, suggesting that metabolism regulates NLRP3 inflammasome activity in fungal infections.

## Introduction

The innate immune system plays important roles in recognising and responding to pathogenic microbes, so that infections are prevented, controlled and resolved. A key immune pathway that responds to infection is orchestrated by the NLRP3 inflammasome [1, 2]. Activation of the cytoplasmic NOD-like receptor NLRP3 by microbial invasion of macrophages results in the assembly of a higher-order inflammasome complex that contains oligomers of NLRP3, the adaptor protein ASC and the protease caspase 1, reviewed in [3]. Assembled inflammasome mediates auto-proteolytic activation of caspase 1, which in turn leads to two functional consequence: (i) cleavage of the inflammatory cytokines IL-1β and IL-18 to their active versions, and (ii) programmed cytolysis of macrophages called pyroptosis, which is mediated by cleavage of gasdermin D and formation of gasdermin D pores in the plasma membrane [4–9]. These caspase 1-driven events are highly pro-inflammatory and orchestrate the immune response by producing and releasing bioactive cytokines, expelling intracellular pathogens and recruiting and activating neutrophils to promote microbial clearance [1, 10].

Potent inflammation can lead to tissue destruction and pathology. Therefore, macrophages have to carefully regulate the activity of NLRP3. In the context of infection, this means that the NLRP3 pathway needs to respond to dangerous microbial colonisation (i.e. infection), but there should be limited activation in response to benign, harmless colonisation. Indeed, while NLRP3 inflammasome contributes to host defences against microbial pathogens [11–15], if not tamed, hyper-inflammatory pathology can actually drive disease [15–21]. Our understanding of how the NLRP3 infammasome is controlled during infection remains incomplete, particularly since there is little evidence that NLRP3 binds to microbial ligands directly. Instead, NLRP3 is a sensor of physiological disturbances, such as potassium efflux, increased levels of reactive oxygen species (ROS) and breaches of plasma membrane integrity that result from microbial invasion or other dangers [3, 22]. Although the precise mechanisms remain to be understood, these “signals” are thought to lead to a conformational change in NLRP3 that initiates assembly of the inflammasome complex [23].

An important human pathogen recognised by the NLRP3 inflammasome is the yeast *Candida albicans*. Normally a benign coloniser of the human gut and mucosal surfaces, *C*. *albicans* is also an opportunistic pathogen causing oral, genital and invasive infections [24]. Precise regulation of NLRP3 activity is thought to be critical in fungal infections and it needs to be carefully balanced. In fact, both protective and pathological roles for NLRP3 inflammasome-driven inflammation have been found in models of *C*. *albicans* infection [11–13],[17–20]. Yet, precisely how macrophages regulate NLRP3 when responding to *C*. *albicans* is unclear.

It has been suggested that the NLRP3 inflammasome responds to virulence traits of *C*. *albicans*, and is activated when *Candida* transitions from the yeast morphotype (which is less invasive and more aligned with the benign state), to the invasive hyphal morphotype (which causes tissue damage and promotes pathogenesis) [25, 26]. Proposed mechanisms by which fungal hyphae could be sensed by the inflammasome are breaching of the phagosome membrane resulting from hyphal growth (but this has recently been disputed [27]), expression of a hyphae-specific membrane-lysing toxin or *via* recognition of cell surface properties that differ between yeast and hyphal cells [2, 11, 25, 26, 28–32]. The problem with this model is that recent work has uncoupled the strict requirement for hyphae in inflammasome activation. First, hyphae are not necessary to trigger caspase 1-dependent pyroptotic cell death of macrophages (*C*. *albicans* mutants locked in yeast form can do it) [31]. Second, hyphae are also not sufficient to trigger inflammasome activation, as there are mutants that can form hyphae but are compromised in causing pyroptosis and IL-1β production [32–34].

Recent work has put macrophage immunometabolism centre stage in infectious diseases [35]. Upon sensing of microbes, major reprogramming of macrophage metabolism leads to upregulation of glycolysis, increased glucose consumption and accumulation of TCA cycle intermediates, which together drive pro-inflammatory antimicrobial responses [36–38]. While these metabolic mechanisms are thought to promote clearance of infection, we have recently identified that microbial pathogens can fight back, taking advantage of the metabolic reprogramming of macrophages and turning it into a weakness [39]. In *C*. *albicans*-infected macrophages, the pathogen competes with immune cells for glucose and triggers glucose starvation and massive macrophage cell death [39]. The exact consequence of glucose starvation and macrophage killing by *C*. *albicans* remains to be understood. In other words, is this an immune evasion mechanism by *C*. *albicans*? An alternative possibility is that this constitutes a host response, by which macrophage glucose starvation and cell death regulate immune responses are needed to control *C*. *albicans*. These two possibilities are not mutually exclusive–*Candida* might try to evade immunity *via* glucose competition, which is then sensed by macrophages to trigger protective responses.

Here we demonstrate that the major consequence of *Candida*-induced glucose starvation in macrophages is activation of the NLRP3 inflammasome. We determine the mechanistic requirements for metabolic activation of the inflammasome by *C*. *albicans* and show that the metabolic mechanism of inflammasome activation is broadly conserved in diverse *C*. *albicans* clinical isolates. Our study demonstrates that increased pathogen load leading to glucose starvation is the central signal for immune cells to trigger inflammatory responses to *C*. *albicans*, while recognition of “virulence factors” in the form of hyphae plays a more restricted role. Taken together, our results show that metabolic competition between macrophages and *C*. *albicans* results in inflammasome responses, indicating that macrophage killing by glucose starvation triggers host protective processes. Sensing metabolic homeostasis as a mechanism for controlling immune activation is likely to be broadly relevant to microbial pathogenesis.

## Results

### Glucose starvation is the dominant mechanism of macrophage killing by diverse *C*. *albicans* clinical isolates, while only some isolates trigger robust pyroptosis

**Fig 1:**
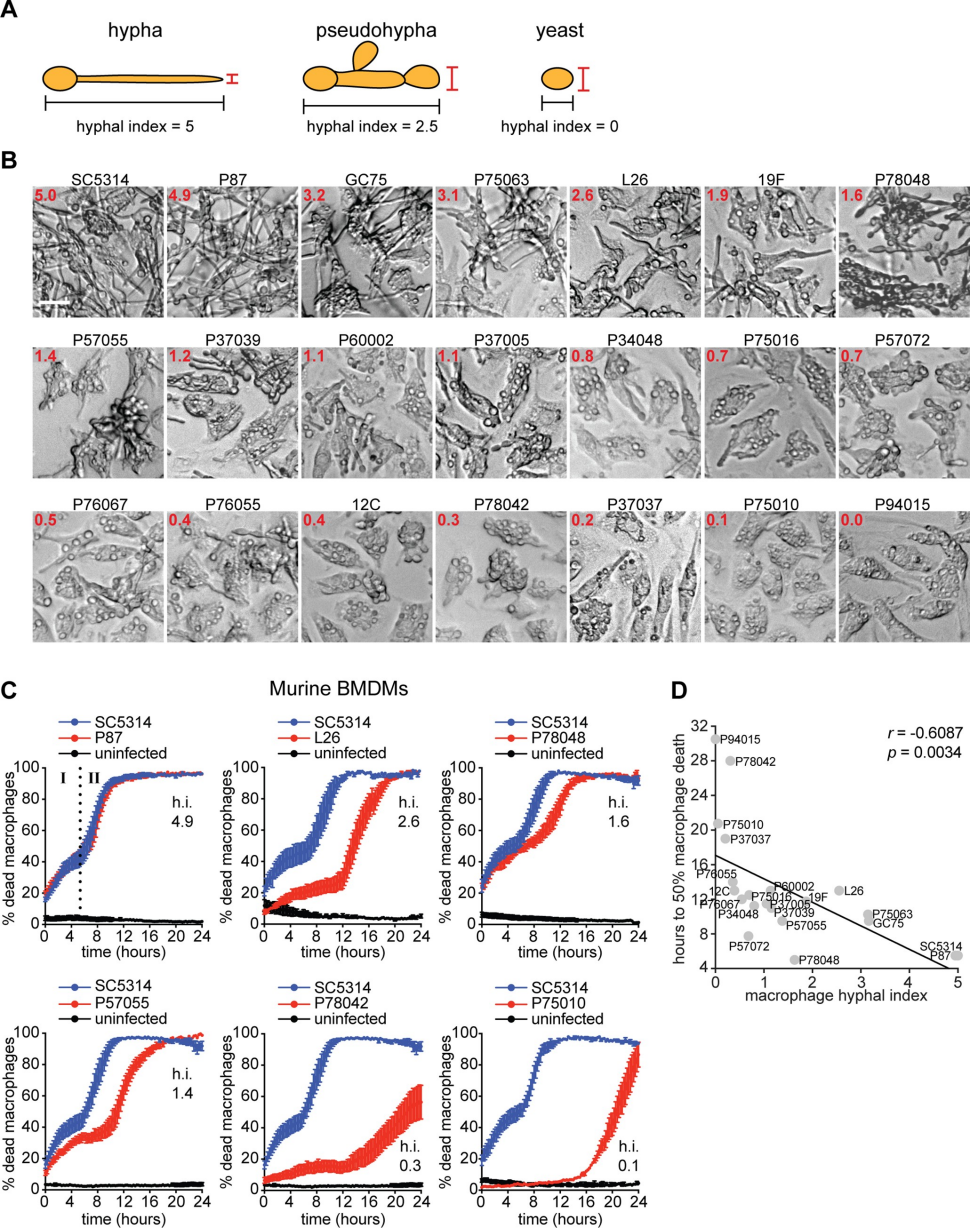
Clinical *C*. *albicans* isolates show different inflammasome-dependent killing of macrophages, but broadly conserved killing by glucose starvation. (A) Illustration of hyphal index calculation. For a given cell, the length from end-to-end (including all connecting septal junctions) was measured and divided by the width–defined as the widest measurement at the distal tip. True hyphae have a large hyphal index (great in length, narrow in width); pseudohyphae are reduced in length and greater in width; and yeast cells have a low hyphal index (similar proportions for both length and width). (B) Microscopy images of *C*. *albicans* clinical isolates 3 h post-phagocytosis in murine bone marrow-derived macrophages (BMDMs). The MOI was 6 *Candida* to 1 macrophage. Values in top-left corner of each image indicate the hyphal index, with the benchmark strain SC5314 normalized to 5. For each isolate, 200 cells were measured. Scale bar is 20 μm. These same images are also displayed in S1A Fig for direct comparison of hyphal index in the same tissue culture media but without macrophages. (C) BMDMs were infected with *C*. *albicans* strains at 6:1 MOI and assessed for cell death by live cell imaging over 24 h. Each graph compares a clinical isolate with the benchmark strain SC5314 and uninfected macrophages control, all assayed in the same live cell imaging experiment (note that P57055 and P78042 were assayed in the same experiment and therefore are being compared to the same control data (i.e. the data for strain SC5314 and the uninfected control is the same in these two graphs; the isolates are displayed in separate graphs for clarity in presentation). Data are the mean values and SEM from 2 independent experiments involving 4 different colonies (each colony was treated as a biological replicate) of the indicated *Candida* strain and at least 2000 macrophages surveyed for each strain, per experiment. The distinction between Phase I (inflammasome dependent killing) and Phase II (glucose starvation-dependent killing) is indicated in the first graph (labeled “I” and II”). The hyphal index (h.i.) is indicated for the clinical isolate being compared to SC5314 (SC5314 has an index of 5). A selection of clinical strains is shown here, and the entire set is displayed in S3 Fig. (D) Correlation plot of the time (in hours) until 50% macrophage death was reached versus hyphal index in macrophages, with the Pearson’s correlation coefficient (*r*) and *p* values.

Following infection in vitro, *C*. *albicans* kills the entire macrophage population even at a low multiplicity of infection. This process encapsulates two key facets of *C*. *albicans*-macrophage interactions: NLRP3 inflammasome activation and competition for glucose between host and pathogen [32, 33, 39]. After infection with *C*. *albicans*, the first wave of macrophage death is driven by activation of NLRP3 inflammasome-dependent pyroptosis and has been termed “Phase I death” [32, 33]. The second wave is due to *C*. *albicans* starving macrophages of essential glucose [39], and has been termed “Phase II death” [32]. The two phases of macrophage death are depicted in Fig 1C, top left panel.

Our current understanding of *C*. *albicans*-induced macrophage death comes predominantly from studying a prototype clinical isolate called SC5314 and laboratory strains derived from it. However, patients are infected with their own commensal strains of *C*. *albicans*, which display great phenotypic diversity [40]. To overcome this limitation, we used live cell imaging to systematically analyse macrophage death with SC5314 and 20 other phylogenetically diverse strains, which originate from a number of body sites in patients and healthy individuals (oral, bloodstream and vaginitis) [40] (S1 Table).

First, we performed a set of control experiments to compare key phenotypes that could affect the ability of *C*. *albicans* isolates to kill macrophages: hyphal formation, growth rates and phagocytosis rates. Hyphal formation was assessed in tissue culture medium and during infection of bone marrow-derived macrophages (BMDMs). This set of strains has been profiled for hyphal formation *in vitro* [40], but they have not been assessed during in macrophages. A hyphal index was calculated after 3 h of infection by dividing the total length of hyphae with the width at the broadest point along the hyphal tip, and then normalizing to give a ratio. A hyphal index of 5 corresponds to true hyphae, while a hyphal index approaching 0 represents primarily yeast cells (Fig 1A). The isolates exhibited a range of filamentation phenotypes in macrophage co-incubations, with only some able to form robust hyphae and many showing more modest to low hyphal indexes (Fig 1B). Of the 21 strains, only SC5314 and P87 showed rapid, near-uniform true hyphae formation across the entire population of macrophages. Overall, there was a high level of correlation of the hyphal index in macrophages with the hyphal index during growth *in vitro* in liquid tissue culture media (see S1A Fig for images and S1B Fig for the correlation plots). Exceptions were strains P60002, P76067, P37037, and P76055, which were capable of forming substantial hyphae in liquid media, but were poor in macrophages, with most of the cells remaining trapped in macrophages after 3 h (Fig 1B and S1A Fig). These outliers tend to have slow doubling time *in vitro*, as measured by growth at 37°C in minimal media using glucose or the non-fermentable carbon source glycerol (note that the phagosome is likely glucose poor) (S1C Fig and S1D Fig). Using a selection of strains, growth rates determined by measurement of optical density mainly correlated with metabolic activity measured by the XTT assay (S1E Fig). There was no correlation of doubling time with hyphal formation *in vitro* (S1C Fig and S1D Fig). However, there was a significant correlation of doubling time in glucose with hyphal formation during macrophage infections (S1F Fig), although no significant correlation was found with doubling time in glycerol which was much slower for all strains compared to glucose (S1G Fig). While it is not known what the growth of these strains is in the phagosome, our data suggests that macrophages are capable of restraining hyphal formation of diverse *C*. *albicans* strains, particularly those which are slower growing. Phagocytosis of the strains was robust, and there were no major differences in macrophage infection rates (S2A Fig and S2B Fig, 10 strains with a range of hyphal indexes were analysed). Collectively, our data show that hyphal formation in macrophages, the key invasive and pathogenic phenotype thought to drive NLRP3 activation and macrophage killing, is highly variable between *C*. *albicans* clinical strains.

Next, we asked how these strains kill macrophages. There was a strong inverse correlation between the hyphal index in macrophages and the time taken for 50% of macrophage killing to occur (Fig 1D). In other words, strains with a high hyphal index tend to kill macrophages faster due to their ability to trigger a steeper, more rapid Phase I death (see Fig 1C for a selection of strains and S3 Fig for all strains). We have previously shown that around 50% of Phase I death is attributable to inflammasome-dependent pyroptosis [32]. There was a correlation between growth rates of the strains *in vitro* with the speed of macrophage killing, but this was not statistically significant (S2C Fig). Some outliers were also evident. For example, P78048 had a relatively low hyphal index (Fig 1B and S1A Fig), yet it was capable of killing macrophages as fast as the robustly hyphal strain SC5314 during Phase I (Fig 1C). Our analysis further revealed that several *C*. *albicans* strains were very poor or incapable of triggering pyroptotic Phase I death altogether (e.g. P37037, P75010, P78042) (Fig 1C and S3 Fig). Therefore, the ability to trigger inflammasome-dependent pyroptosis is highly variable between *C*. *albicans* clinical strains, questioning the broad relevance of this process in *C*. *albicans*-macrophage interactions.

In contrast, all clinical strains, including those that form poor hyphae, were able to trigger robust glucose starvation-dependent Phase II death (Fig 1C and S3 Fig), showing that glucose starvation is a broadly conserved mechanism of death for *C*. *albicans*-infected macrophages. The timing of Phase II death differed between the strains, but once it started, it proceeded with fast kinetics (Fig 1C and S3 Fig). This means that all strains show some level of escape from macrophages, which likely enables glucose depletion during replication of *C*. *albicans* in the extracellular infection environment. Even strain P94015, which cannot form hyphae, could occasionally escape from macrophages sufficiently to kill the entire population, although this took well over 24 h to occur (S1 Movie).

**Fig 2:**
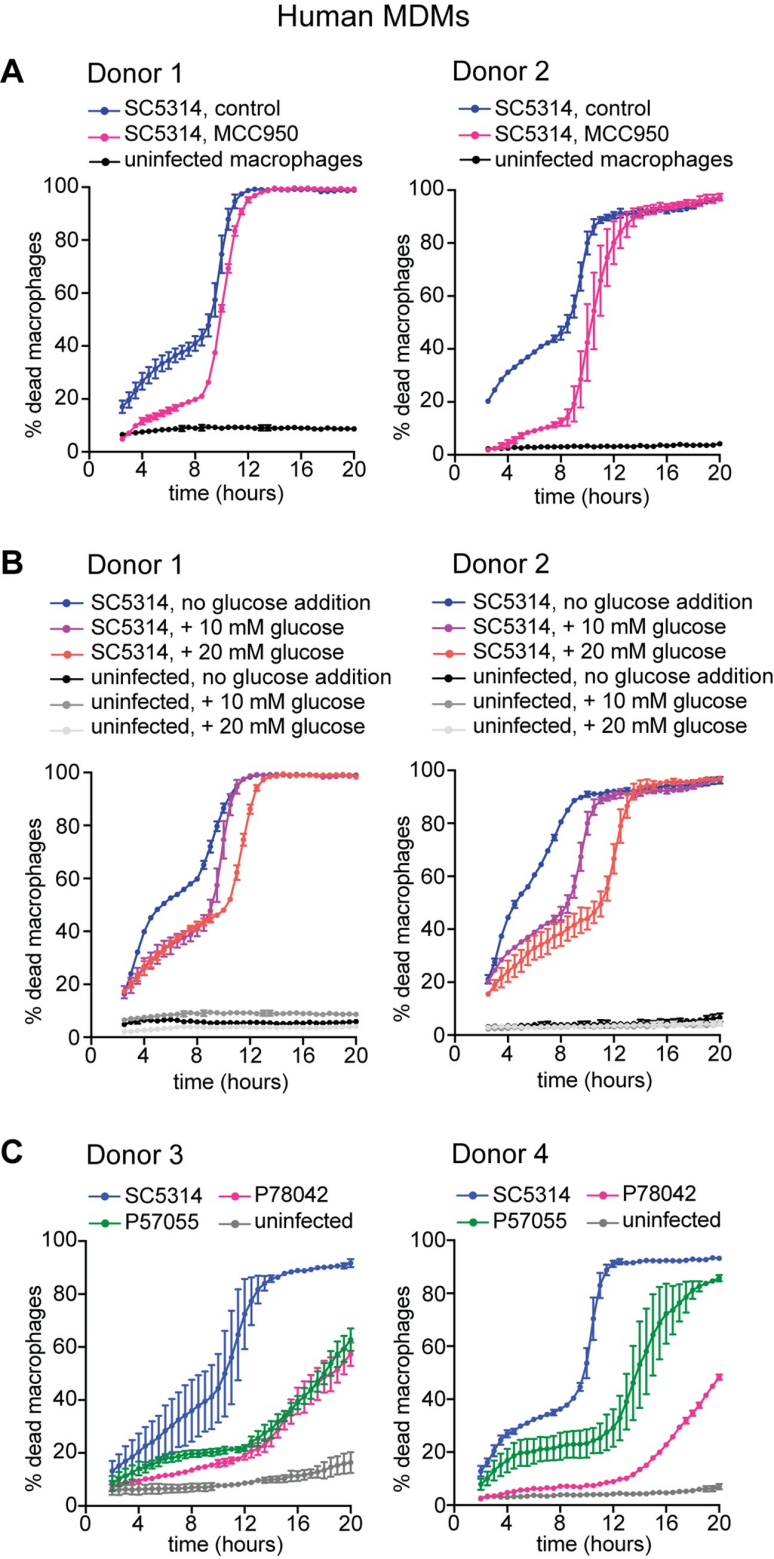
Distinct inflammasome and glucose-dependent killing by *C*. *albicans* isolates is recapitulated in human primary macrophages. (A) Primary human monocyte-derived macrophages (hMDMs) infected with *C*. *albicans* strain SC5314 at 6:1 *Candida*:macrophage MOI. hMDMs were collected from two donors and were assayed on the same day in the same experiment. The two donors are plotted separately. Each graph shows the average and SEM of two technical repeats. At least 1500 macrophages were counted per condition, per donor. The NLRP3 inhibitor MCC950 was added at 10 μM to assess the involvement of NLRP3 inflammasome-dependent pyroptosis during Phase I death of *Candida*-infected hMDMs. (B) Same experiment as in A, this time comparing media with distinct glucose concentrations to assess glucose starvation during Phase II death of hMDMs. All of the conditions (+/-MCC950 shown in A and medium with increasing glucose concentrations shown in B) were assayed together in the same experiment but are plotted separately for clarity. Therefore, data for uninfected controls and infections in medium with 10 mM glucose are the same in panel A and panel B for each donor. (C) hMDMs infected with *C*. *albicans* strains SC5314, P57055 and P78042 at 6:1 *Candida*:macrophage MOI. hMDMs were collected from two different donors and are plotted separately. Each graph shows the average and SEM of two technical repeats. At least 1500 macrophages were counted per condition, per donor.

Using strain SC5314, we show that the two mechanisms of macrophage cell death, namely NLRP3-dependent pyroptosis and glucose starvation-dependent death, are recapitulated in primary human monocyte-derived macrophages (hMDMs) and follow similar kinetics (Fig 2A and 2B). The NLRP3 inhibitor MCC950 rescued hMDMs from *C*. *albicans*-induced Phase I death, showing NLRP3-dependent pyroptosis (Fig 2A). Glucose supplementation delayed Phase II death of hMDMs, showing glucose starvation-dependent death (Fig 2B). As in mouse BMDMs, only some *C*. *albicans* strains triggered inflammasome-dependent pyroptosis of hMDMs depending on their hyphal index: SC5314 (hyphal index 5) triggered robust inflammasome-dependent pyroptosis, P57077 (hyphal index 1.4) triggered more modest pyroptosis and P78042 (hyphal index 0.3) did not trigger pyroptosis (Fig 2C). In contrast, all three strains triggered glucose starvation-dependent Phase II death of hMDMs (Fig 2C).

### *C*. *albicans* triggers inflammasome activation in two temporally distinct events

Given that several *C*. *albicans* strains failed to induce robust inflammasome-dependent Phase I killing of macrophages (Fig 1), the next question was whether all *C*. *albicans* isolates are recognised by macrophages to activate the inflammasome. To answer this, we imaged inflammasome activation over time using immortalized BMDMs expressing fluorescently labelled ASC, the adaptor subunit of the NLRP3 inflammasome (ASC-mCerulean) [41]. Activation of the inflammasome complex is associated with oligomerization of ASC and the formation of a visible large “speck” (~1 μm), which allows for quantification of inflammasome activation events in macrophage populations over time using live cell imaging. In parallel, we examined host mitochondrial health by staining mitochondria with TMRM (signal intensity depends on mitochondrial membrane potential), and we also quantified macrophage cell death. The live cell imaging results for all 21 *C*. *albicans* isolates, along with the positive control nigericin (a potent inducer of the NLRP3 inflammasome) and the uninfected negative control are shown in S4 Fig.

Unexpectedly, all *C*. *albicans* strains triggered inflammasome activation (S4 Fig), even those that formed poor or no hyphae and could not trigger “Phase I” inflammasome-dependent pyroptotic death in macrophages (Fig 1). Interestingly, depending on morphological phenotype and infection loads, the kinetics and mechanisms of inflammasome activation were distinct. Below we describe our conclusions by comparing the prototype highly hyphal strain SC5314 and a selected bloodstream infection isolate with poor hyphae, P78042.

**Fig 3:**
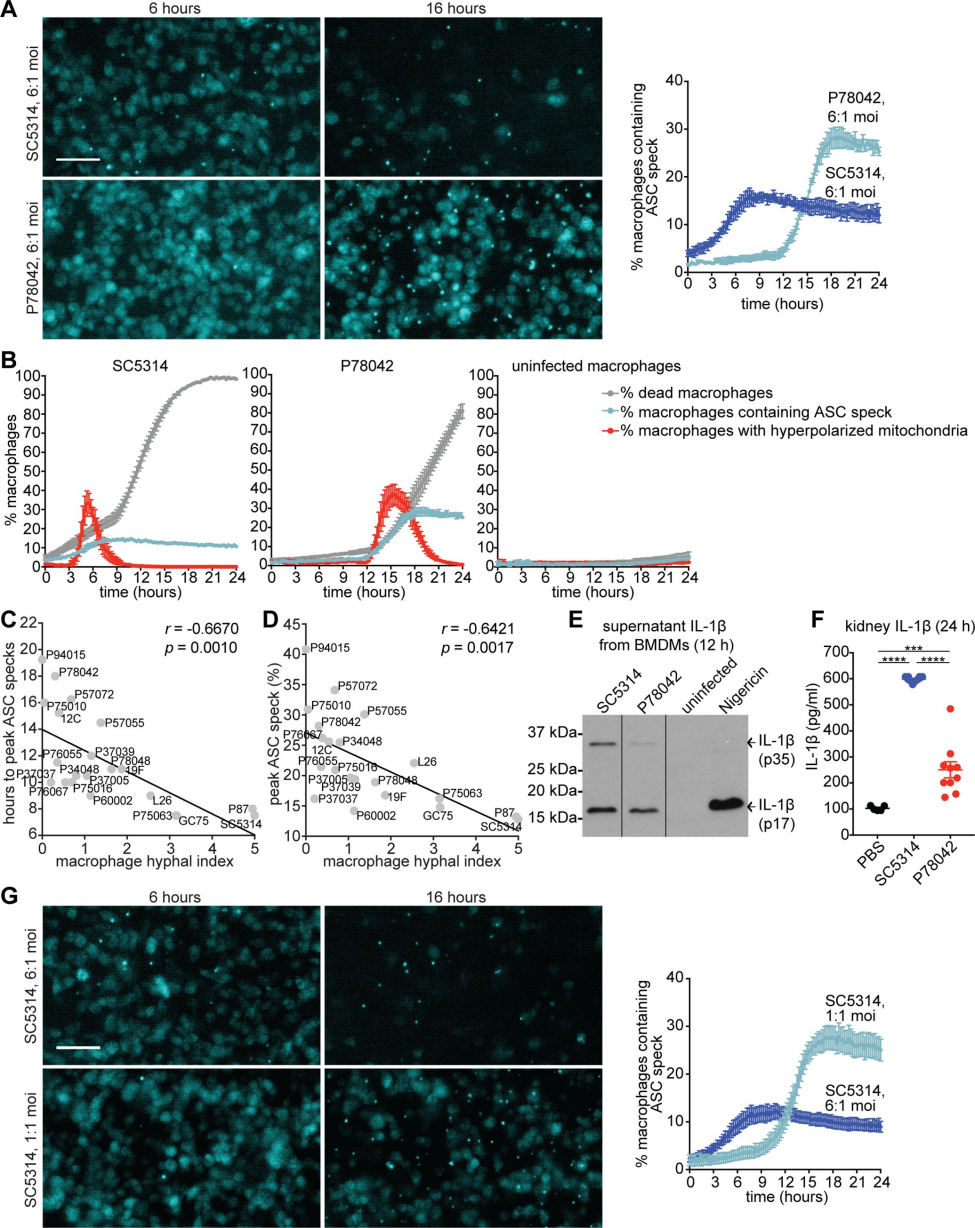
Inflammasome activation in response to *C*. *albicans* occurs in two temporally distinct events. (A) ASC-mCerulean-expressing immortalized macrophages following challenge with *Candida* strains SC5314 or P78042 at MOI 6:1. Left panel: representative images from live cell microscopy at 6 and 16 h post-phagocytosis, with ASC-mCerulean detection displayed in cyan. Scale bar is 50 μm. Right panel: graph showing quantification of ASC speck formation over time. Data are the mean values and SEM from 4 independent experiments. (B) Experimental conditions as in A. Shown are % macrophages containing an ASC speck, % macrophages with hyperpolarized mitochondria and % dead macrophages quantified in live cell imaging experiments. ASC speck data is the same as in panel A. Data are the mean values and SEM from 4 independent experiments (see S4 Fig for complete set of data involving all 21 clinical isolates). (C) Correlation plot of the time post-phagocytosis when peak ASC speck formation occurs versus hyphal index of the *Candida* isolates in macrophages, with the Pearson’s correlation coefficient (*r*) and *p* values as indicated. (D) Correlation plot of peak percentage of macrophages in the population that contain an ASC speck versus hyphal index in macrophages, with the Pearson’s correlation coefficient (*r*) and *p* values as indicated. (E) IL-1β detection following challenge with *Candida* strains SC5314 or P78042 at MOI 6:1. Primary murine BMDMs were primed with LPS (50 ng/ml) for 3 h, followed by challenge with *Candida* strains SC5314 or P78042 (MOI 6:1), nigericin (10 μM), or left uninfected. At 12 h post-challenge, supernatants were analysed by immunoblot for IL-1β. Shown is one representative immunoblot from two independent experiments involving different mice. Note that this immunoblot contains additional lanes that were spliced out (indicated by the black lines); see S5 Fig for the entire, uncropped immunoblot from this experiment. (F) BALBc mice were injected with 1x10^6^ CFU of the indicated *C*. *albicans* strains: SC5314 (highly hyphal, triggers the early wave of inflammasome activation) or P78042 (poorly hyphal, triggers only the second wave of inflammasome activation). Mock injection was with PBS. Levels of IL-1β in kidney were measured by ELISA (n = 10 animals/group for *C*. *albicans* infection, and 5 for PBS injection). *** *p* < 0.001, **** *p <* 0.0001, Student’s two-tailed *t* test. (G) ASC speck formation over time in infections with the highly hyphal *C*. *albicans* strain SC5314 at high (6:1) and low (1:1) MOI. Left panel: representative images from live cell microscopy at 6 and 16 h during infection, with ASC-mCerulean detection displayed in cyan. Scale bar is 50 μm. Right panel: graph showing quantification of ASC speck formation over time. Data are the mean values and SEM from 5 independent experiments.

In the presence of robust hyphae (strain SC5314), the bulk of the inflammasome activation events (i.e. ASC speck formation) occurred early, in the first 6 h following the start of infection (Fig 3A). The peak of ASC speck-positive cells in the population was ~15% by 6 h, after which it plateaued. ASC speck formation was preceding cell death (typically ~15–30 minutes before), consistent with activation of inflammasome-dependent pyroptosis during Phase I death of macrophages. This data is consistent with our previous study using a laboratory strain derived from SC5314 [42]. Similar to our previous finding with primary macrophages (BMDMs) [39], *C*. *albicans* infection of the ASC-mCerulean immortalised BMDMs triggered hyperpolarisation of macrophage mitochondria after several hours of infection preceding the rapid Phase II death (Fig 3B). This indicates that macrophages are experiencing mitochondrial dysfunction. During the early, Phase I activation of the inflammasome some ASC specks were occurring well in advance of mitochondrial hyperpolarization, but additional ASC specks formed coinciding with mitochondrial hyperpolarisation (Fig 3B).

A completely different scenario was seen with the poorly hyphal strain P78042. There was essentially no ASC speck formation in the first 6 h post infection; instead a rapid increase in ASC specks started at around 12 h post infection indicating robust inflammasome activation (Fig 3A). This later event of inflammasome activation strongly coincided with hyperpolarization of macrophage mitochondria and the initiation of the rapid Phase II death in macrophage populations (Fig 3B). In most instances, the later ASC speck formation also preceded the appearance of DRAQ7 staining, which occurred 1–2 frames later (corresponding to a 15–30 minutes lag). The later, Phase II event of inflammasome activation was more pronounced than the early Phase I wave. When challenged with P78042, ~30% of macrophages contained an ASC speck at the peak time point, compared to ~15% for early activation seen with SC5314 (Fig 3B; of note, the vast majority of ASC specks persisted and remained clearly detectable after cell death and throughout the rest of the imaging). The conclusion that less hyphal isolates trigger later, but more potent inflammasome activation broadly held when analysing the entire set of 21 clinical strains: hyphal index was inversely correlated to the time needed to reach the peak number of macrophages in the population displaying an ASC speck (Fig 3C), and also inversely correlated to the maximal percentage of macrophages in the population displaying an ASC speck at the peak time point (Fig 3D). To ascertain that this newly discovered Phase II wave of inflammasome activation is functionally relevant, we asked whether it led to the key functional output of IL-1β maturation. Indeed, that was the case—the active (17 kDa) peptide was detected 12 h following challenge of macrophages with strain P78042, which can only trigger Phase II inflammasome activation (Fig 3E and see S5 Fig for uncropped Western blot). P78042 also induced substantial levels of IL-1β in infected kidneys in the murine candidaemia model (Fig 3F), showing relevance of this process in animal infection *in vivo*.

The macrophage experiments described so far were done using a multiplicity of infection (MOI) of 6 *Candida* to 1 macrophage. We wondered if, in addition to hyphal morphology, fungal load impacted on the kinetics of inflammasome activation. To test that, we infected macrophages with the highly hyphal strain SC5314 at both high MOI of 6 and low MOI of 1. This experiment revealed that the early, Phase I event of inflammasome activation is only seen with MOI 6, i.e. when there is a large number of fungal hyphae infecting macrophages (Fig 3G). With MOI of 1, only the later, Phase II event of inflammasome activation was seen even though SC5314 forms robust hyphae (Fig 3G).

### Mechanistic requirements for Phase II inflammasome activation

Studies so far have focused on the early, Phase I activation event to identify hyphal virulence determinants and cell surface components of *C*. *albicans* that are sensed by the NLRP3 inflammasome [25, 27, 31–33, 42]. We now show that this early event is only seen under a specific set of experimental conditions, i.e. only with a subset of clinical strains of *C*. *albicans* which depending on hyphal morphology and when a high initial MOI is used. Instead, the second, Phase II event of inflammasome activation is the predominant mechanism in response to diverse *C*. *albicans* isolates (Fig 3 and S4 Fig), suggesting that NLRP3 activation does not critically depend on defined fungal cell morphology or surface structure. We subjected macrophages to a series of chemical treatments to understand what is driving the second inflammasome activation mechanism and compared this to the early mechanism as a control.

**Fig 4:**
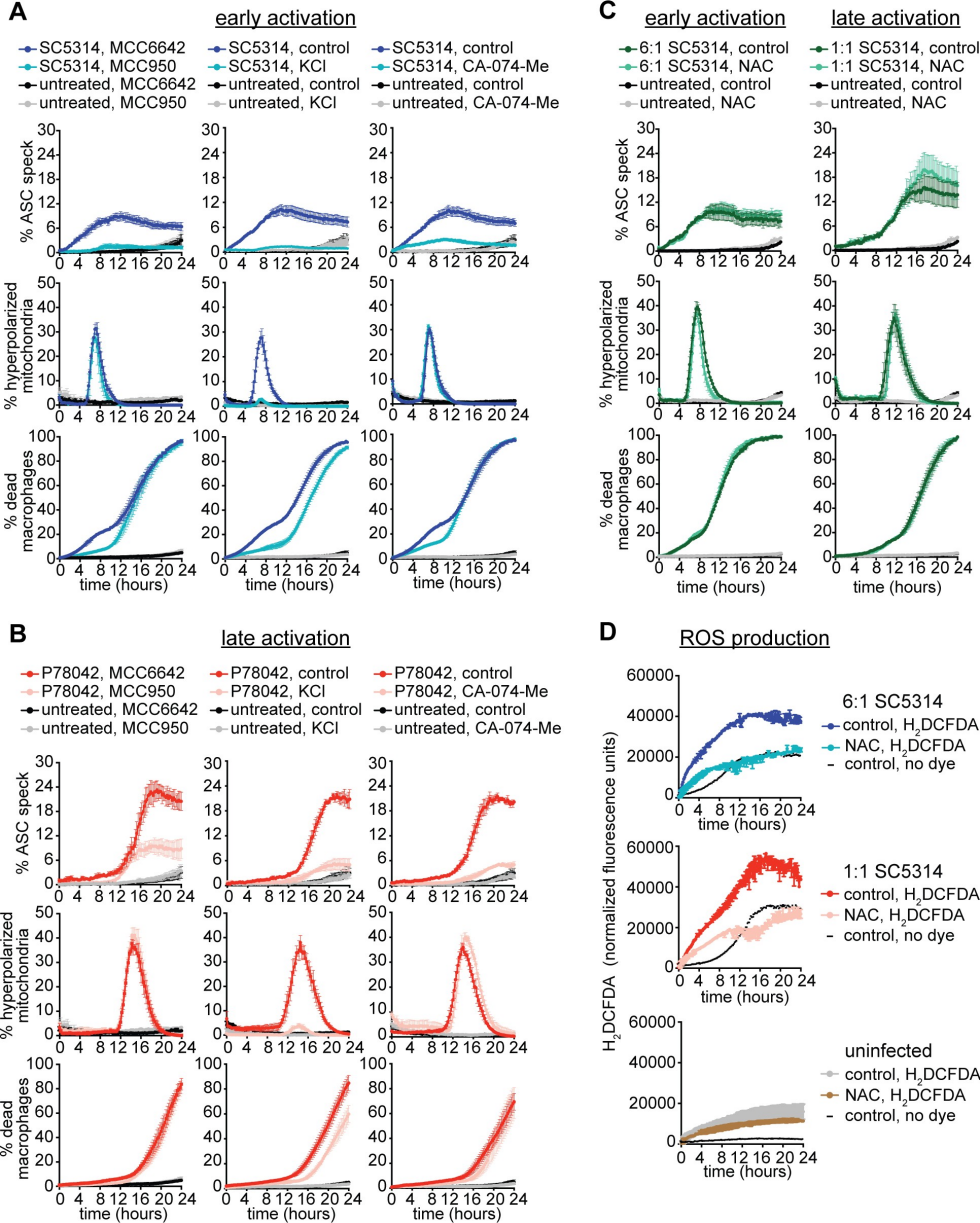
The second mechanism of inflammasome activation depends on NLRP3, potassium efflux and release of the lysosomal protease cathepsin B. (A) To assay the early event of inflammasome activation, ASC-mCerulean-expressing immortalized macrophages were challenged with *Candida* strain SC5314 (MOI 6:1). Uninfected macrophage controls were also included. The % macrophages containing an ASC speck, % macrophages with hyperpolarized mitochondria and % dead macrophages were quantified in live cell imaging experiments. Comparisons were assessed in the presence of the NLRP3 inhibitor MCC950 (10 μM) or the control inactive compound MCC6642 (10 μM), in the presence of cathepsin B inhibitor CA-074-Me (10 μM) or control (DMSO solvent) and in the presence of KCl (30 mM) or control (water solvent). Data are the mean values and SEM from 3 independent experiments involving at least 6000 macrophages surveyed for each condition, per experiment. (B) Same experimental setup as described in (A), but to assay the later event of inflammasome activation *C*. *albicans* strain P78042 was used (MOI 6:1). Data are the mean values and SEM from 3 independent experiments involving at least 6000 macrophages surveyed for each condition, per experiment. (C) Same experimental setup as described in (A). Macrophages were challenged with *Candida* strain SC5314 (MOI 6:1 and 1:1) or left uninfected, in the presence of the ROS scavenger N-acetyl-L-cysteine (NAC; 5 mM) or control (PBS solvent). Data are the mean values and SEM from 3 independent experiments involving at least 6000 macrophages surveyed for each condition, per experiment. Note that the 6:1 and 1:1 MOI infections were assayed in the same experiments alongside untreated controls; therefore, the data for “untreated, control” and “untreated, NAC” (black and grey lines) is the same in the left and right panels. The data for the two MOIs are displayed in separate graphs for clarity in presentation. (D) Quantification of ROS production in primary murine BMDMs. Macrophages were pre-loaded with 20 μM H_2_DCFDA (or no dye control) for 30 min and then infected with *C*. *albicans* SC5314 (MOI 6:1 or 1:1), or left uninfected, in media containing 5 mM antioxidant N-acetyl-L-cysteine (NAC) or control (water). NAC treatment significantly reducing ROS accumulation during *C*. *albicans* infections. Data are the mean values and SEM from 2 independent experiments.

During early inflammasome activation ASC speck formation could be inhibited with the NLRP3 inhibitor MCC950 [43], the cathepsin B inhibitor CA-074-Me and by inhibiting potassium efflux with a high concentration of KCl (Fig 4A). All these treatments also inhibited the pyroptotic, Phase I death of macrophages (Fig 4A). These results are broadly consistent with published work by us and others on the requirements for early, Phase I NLRP3 inflammasome activation and pyroptosis by *C*. *albicans* [25, 27, 42]. Phase II inflammasome activation was also substantially inhibited by the NLRP3 inhibitor MCC950, and even more so by KCl and the cathepsin B inhibitor CA-074-Me, although some residual ASC speck formation was seen in the presence of these chemicals (Fig 4B). KCl reduced mitochondrial hyperpolarisation that was occurring with Phase II inflammasome activation, but MCC950 and CA-074-Me did not (Fig 4B). This indicates that MCC950 and CA-074-Me act down-stream of mitochondrial dysfunction to block inflammasome activation by *C*. *albicans*. Although MCC950, KCl and CA-074-Me all substantially reduced Phase II ASC speck formation during infections with strain P78042, none of these chemicals had a major effect on macrophage cell death (Fig 4B). These results indicate that Phase II inflammasome activation by *C*. *albicans* does not trigger pyroptosis. This conclusion aligns with our previous results with SC5314 showing that Phase II macrophage death still occurs in *Casp1/11*^*-/-*^ mutant BMDMs [32]. We now show that this conclusion also holds when various inflammasome mutant BMDMs (*Casp1/11*^*-/-*^, *Nlrp3*^*-/-*^ or *Gsdmd*^*-/-*^) are infected with diverse clinical isolates of *C*. *albicans* (S6 Fig). In all cases, inflammasome mutations reduced Phase I death (showing it is driven in part by pyroptosis), but Phase II death occurred and proceeded to completion even if with slightly different kinetics in some of the inflammasome mutants (S6 Fig).

ROS production has been implicated as a trigger of NLRP3 inflammasome activation and linked to mitochondria [44–46]. Given the temporal correlation of mitochondrial hyperpolarisation during *C*. *albicans* infection and the later wave of inflammasome activation, we tested if ROS was involved. However, that did not seem to be the case. The ROS scavenger N-acetyl-L-cysteine (NAC) had no effect on inflammasome activation or macrophage death in infections with the hyphal strain SC5314 at MOIs of 6:1 (reporting on Phase I inflammasome activation) or 1:1 (reporting on Phase II inflammasome activation) (Fig 4C), although it clearly reduced intracellular ROS accumulation as measured in primary macrophages (Fig 4D).

### Phase II inflammasome activation is triggered by macrophage glucose starvation

**Fig 5:**
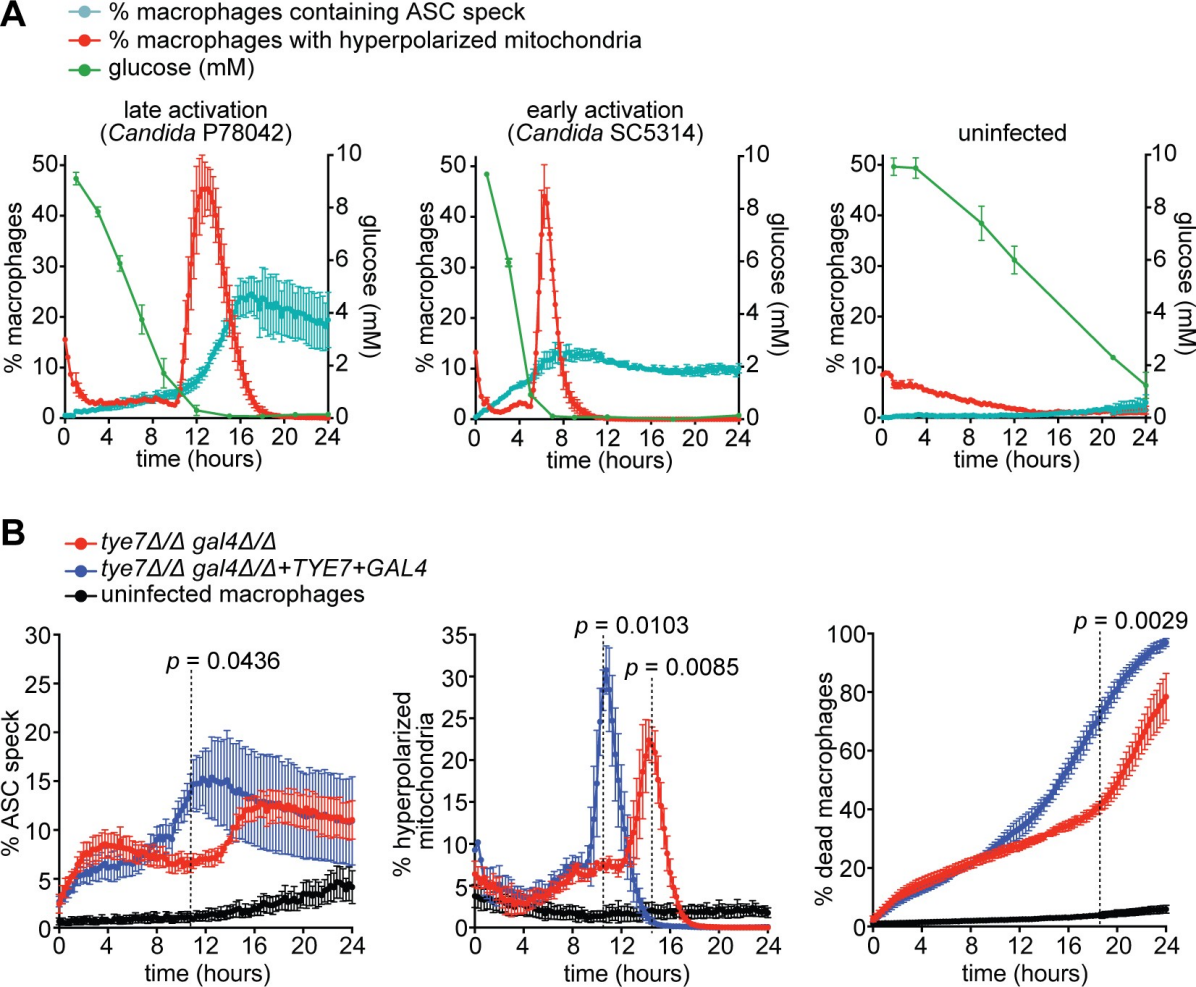
Activation of the inflammasome is triggered by macrophage glucose starvation. (A) ASC-mCerulean-expressing immortalized macrophages following challenge with *Candida* strains P78042 at MOI 6:1 (to assay late activation), SC5314 at MOI 6:1 (to assay early activation), or left uninfected. Plotted on the left axes are % macrophages containing an ASC speck and % macrophages with hyperpolarized mitochondria as quantified in live cell imaging experiments (in cyan and red respectively). Plotted on the right axes are glucose concentration measurements at select time points (in green). Data are the mean values and SEM from 2 independent experiments, and in live cell imaging this involved at least 6000 macrophages surveyed for each condition, per experiment. (B) Comparison of the *C*. *albicans* glycolytic mutant *tye7Δ/Δ gal4Δ/Δ* versus the *TYE7 GAL4* complemented strain in ASC-mCerulean-expressing immortalized macrophages, along with uninfected macrophages (“macrophages only” control). The MOI was 1:1 and starting tissue culture media contained 10 mM glucose. Displayed in each graph are % macrophages containing an ASC speck, % macrophages with hyperpolarized mitochondria and % dead macrophages as quantified in live cell imaging experiments. Data are the mean values and SEM from 3 independent experiments involving at least 10000 macrophages surveyed for each condition, per experiment. *p* values were calculated at the time points indicated by dashed lines (Student’s two-tailed t test).

The Phase II wave of inflammasome activation coincides with hyperpolarisation of macrophage mitochondria (Fig 3 and S4 Fig), which we have shown to be a hallmark of glucose starvation in macrophages during *C*. *albicans* challenge [39]. We therefore hypothesised that the inflammasome is activated by glucose competition between host and pathogen, which results in macrophage glucose starvation, disruption of glycolysis and metabolic dysfunction. Consistent with this hypothesis, during Phase II inflammasome activation (in infection with strain P78042), the majority of ASC specks formed only when glucose levels were below 1 mM (Fig 5A left panel). The reduction in glucose was due to increased microbial load and overlapped with mitochondrial hyperpolarisation in macrophages (Fig 5A left panel). The early, Phase I wave of inflammasome activation (infection with strain SC5314) occurred in the presence of higher glucose and it continued as glucose levels declined and mitochondrial hyperpolarisation initiated (Fig 5A middle panel). Uninfected ASC-mCerulean macrophages consumed glucose more gradually, with a detectable concentration still remaining over the course of 24 h, and no detectable ASC speck formation or cell death over the 24 h time course (Fig 5A, right panel).

Next, we asked if restoring macrophage glucose homeostasis reduces inflammasome activation in response to *C*. *albicans*. First, we probed this prediction by using the *C*. *albicans tye7Δ/Δ gal4Δ/Δ* mutant, which lacks two transcription factors that activate genes of the glycolytic pathway [39, 47]. We have shown previously that *tye7Δ/Δ gal4Δ/Δ* has reduced ability to rapidly compete for glucose with macrophages, leading to slower reduction in glucose levels over time [39]. In response to *tye7Δ/Δ gal4Δ/Δ*, macrophages activated the Phase I wave of ASC speck formation that depends on hyphal formation (Fig 5B, first 4 h post-infection). However, the Phase II wave, which depends on glucose depletion, was delayed (Fig 5B, 4–12 h post-infection). The delay in the second wave of inflammasome activation in response to *tye7Δ/Δ gal4Δ/Δ* correlated with later hyperpolarisation of macrophage mitochondria (the hallmark of glucose starvation) in these immortalised BMDMs, and later initiation of glucose starvation-dependent macrophage death (Fig 5B).

**Fig 6:**
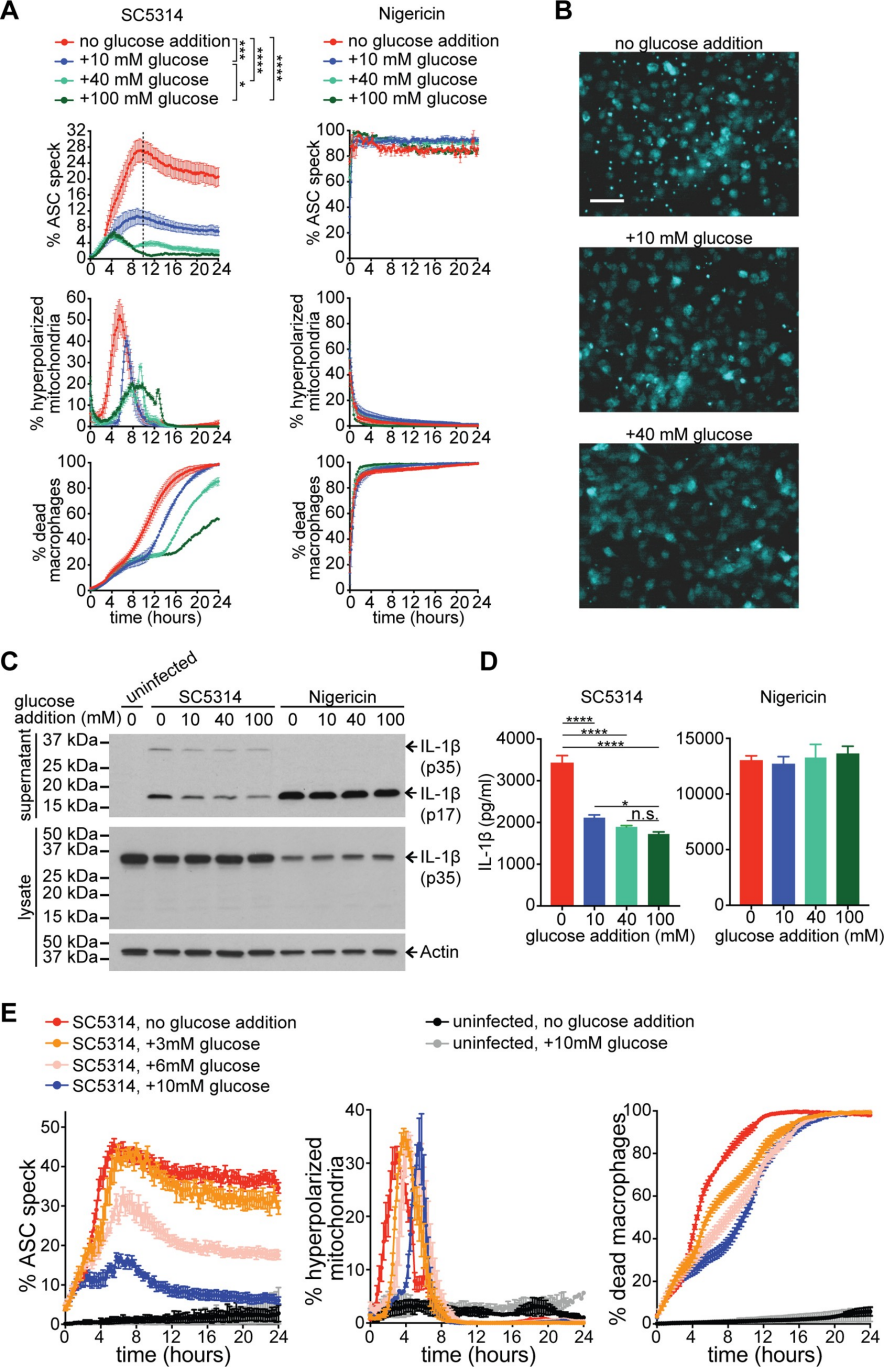
Restoring macrophage glucose homeostasis reduces inflammasome activation. For all experiments in this Figure, the initial tissue culture media contained ~0.65 mM glucose, which was then supplemented with additional glucose as indicated in each panel. (A) ASC-mCerulean-expressing immortalized macrophages following infection with *Candida* hyphal strain SC5314 (MOI 6:1) or nigericin as positive control (10 μM) and with 0, 10, 40, or 100 mM glucose addition. Shown are % macrophages containing an ASC speck, % macrophages with hyperpolarized mitochondria and % dead macrophages, as quantified in live cell imaging experiments. Data are the mean values and SEM from 3 independent experiments. *p* values were calculated for % ASC speck at the time point indicated by dashed line (* *p* < 0.05, *** *p* < 0.001 and **** *P* < 0.0001, one-way ANOVA and Tukey’s multiple comparison test). (B) Representative images from live cell microscopy after 8 h of infection with hyphal strain SC5314 (MOI 6:1) in tissue culture media containing 0, 10, or 40 mM added glucose. ASC-mCerulean detection is displayed in cyan. Scale bar is 50 μm. (C) Primary murine BMDMs were primed with LPS (50 ng/ml) for 3 h, followed by challenge with *Candida* hyphal strain SC5314 (MOI 6:1), nigericin (10 μM), or uninfected. The starting tissue culture media contained 0, 10, 40, or 100 mM added glucose. At 3 h post-challenge, supernatants and lysates were analysed by immunoblot for IL-1β and actin (see S7 Fig for entire, uncropped immunoblots from three independent experiments and the Pounceau staining loading control). (D) The same supernatant samples from the Western blots (displayed in panel C of this Figure and S7 Fig) quantified by ELISA. Data are the mean values and SEM from 3 independent experiments. * *p* < 0.05, and **** *p* < 0.0001, one-way ANOVA and Tukey’s multiple comparison test (n.s.–not significant). (E) ASC-mCerulean-expressing immortalized macrophages following infection with *Candida* hyphal strain SC5314 (MOI 6:1), comparing tissue culture media containing 0, 3, 6, and 10 mM glucose addition. Displayed in each graph are % macrophages containing an ASC speck, % macrophages with hyperpolarized mitochondria and % dead macrophages as quantified in live cell imaging experiments. Data are the mean values and SEM from 2 independent experiments involving at least 9000 macrophages surveyed for each condition, per experiment.

Second, we tested if inflammasome activation can be reduced by supplementing glucose during infection, to alleviate glucose starvation and restore macrophage glycolysis. We first used a high, non-physiological glucose concentration of 100 mM to delay glucose starvation-dependent Phase II killing of macrophages by the hyphae competent strain SC5314 as much as possible, without affecting Phase I inflammasome-dependent killing (Fig 6A). Under this condition, we detected early ASC speck formation associated with Phase I, however there was little inflammasome activation at later time points (Fig 6A). In contrast, ASC speck formation of *C*. *albicans*-infected macrophages was substantially enhanced in low glucose medium (~0.65 mM, denoted as “no glucose addition”), and supplementation of additional glucose (10 or 40 mM) reduced the number of ASC speck-positive macrophages (Fig 6A and images in Fig 6B). Comparing 10 to 40 mM glucose showed that the initial, Phase I wave of ASC speck formation was not affected by higher glucose, but after 4 h ASC speck formation was reduced in medium with higher glucose (Fig 6A). Some ASC specks were re-collapsing back into the cytoplasm at concentrations of glucose of 40 mM, which partially explains the reduced ASC speck detection from 4 to 8 h post-phagocytosis (Fig 6A). The reduction of ASC speck formation by higher glucose concentrations was accompanied by a delayed and more drawn out peak of mitochondrial hyperpolarization and lower levels of death of macrophages (Fig 6A).

To address if the key output of inflammasome activation, IL-1β secretion, was responsive to glucose levels, we used primary BMDMs. While hyphae competent *C*. *albicans* SC5314 was able to induce the NLRP3 inflammasome and IL-1β processing in macrophages in the presence of high concentrations of glucose (100 mM), IL-1β secretion increased in media lacking glucose and this was significantly reduced by 10 and 40 mM glucose (Fig 6C, 6D and S7 Fig). Glucose supplementation did not affect IL-1β secretion and ASC speck formation in nigericin-treated macrophages (Fig 6A, 6C and 6D). The reduction of inflammasome activation by increasing glucose was also clearly observed within a range of physiologically relevant concentrations (Fig 6E; note the progressive drop of ASC speck formation when comparing 3 to 6 to 10 mM glucose). Supplementation with galactose, a carbon source that cannot restore glycolytic homeostasis in macrophages [39], did not reduce ASC speck formation (S8 Fig). We also performed controls for increased osmolarity of high glucose medium by using equivalent concentrations of sorbitol or mannitol instead of glucose. Unlike glucose, neither sorbitol nor mannitol had an effect on ASC speck formation (S8 Fig), showing that the effect of glucose is due to restoring macrophage glucose homeostasis, and is not caused by an indirect effect due to changes in osmolarity in high glucose medium.

### Macrophage glucose starvation is a broadly relevant mechanism for inflammasome activation by diverse *C*. *albicans* isolates

**Fig 7:**
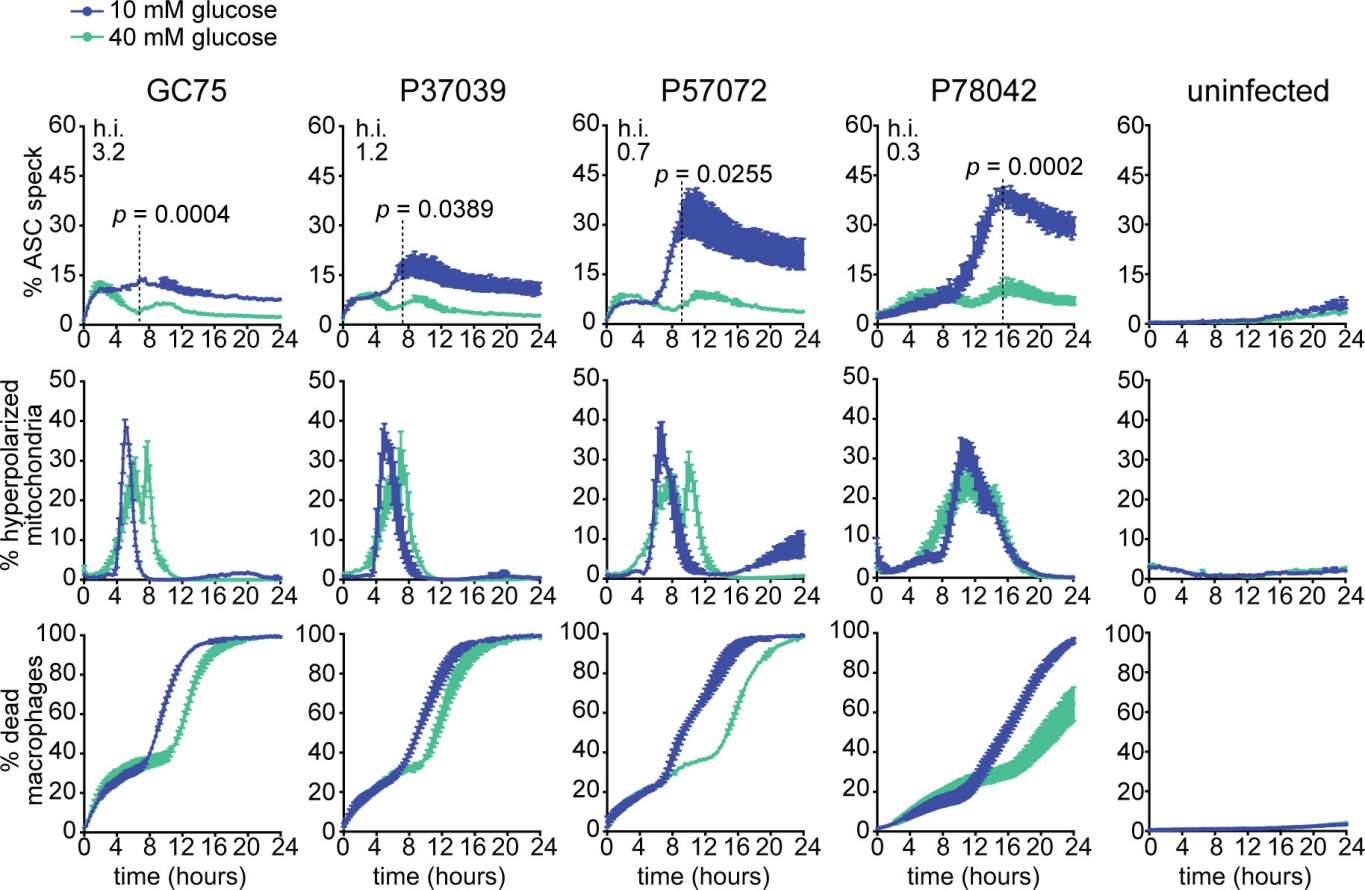
Glucose starvation is a broadly relevant mechanism of inflammasome activation across diverse *C*. *albicans* isolates. ASC-mCerulean-expressing immortalized macrophages were infected with the indicated clinical isolates of *C*. *albicans* at MOI 6:1. Tissue culture media contained 10 or 40 mM glucose. Shown are % macrophages containing an ASC speck, % macrophages with hyperpolarized mitochondria and % dead macrophages as quantified in live cell imaging experiments. Data are the mean values and SEM from 2 independent experiments involving at least 8000 macrophages surveyed for each condition, per experiment. The hyphal index (h.i.) for the *C*. *albicans* strains is shown in the right top corner of the graphs. *p* values were calculated for % ASC speck at the time points indicated by dashed lines (Student’s two-tailed t test).

We next wanted to ascertain that activation of the inflammasome by macrophage glucose starvation is seen not only with SC5314 but is recapitulated with other clinical isolates. Indeed, this was the case. By using an additional panel of *C*. *albicans* clinical strains spanning a variety of hyphal indexes in macrophages (GC75, P37039, P57072 and P78042), we compared ASC speck formation in medium with 10 mM versus 40 mM glucose. In each case, higher glucose levels resulted in reduced numbers of ASC specks, showing reduced activation of the inflammasome (Fig 7 top panel). Moreover, same as for the prototype strain SC5314, supplementation of additional glucose resulted in delayed mitochondrial hyperpolarisation and macrophage cell death in response to the other *C*. *albicans* clinical isolates tested (Fig 7, middle and lower panel). These data support the proposition that infection-induced macrophage glucose starvation is a broadly relevant mechanism of inflammasome activation in response to *C*. *albicans*.

## Discussion

Here we show that *C*. *albicans* triggers activation of the NLRP3 inflammasome by causing glucose starvation in macrophages. This discovery establishes a mechanism of inflammasome activation that is driven by host-pathogen competition for glucose and does not depend on *Candida* hyphal formation. Instead, it critically depends on increased fungal load driving down glucose levels in the infection microenvironment. By sensing the metabolic activity of *C*. *albicans* and responding when their metabolism has been disrupted by glucose starvation, macrophages can detect when *C*. *albicans* colonisation levels are dangerously high, to then activate the NLRP3 inflammasome. At lower colonisation levels, immune cells are likely to be able to control *C*. *albicans* without triggering potent inflammation. This might reflect homeostatic mechanisms to regulate inflammation and avoid tissue pathology.

In macrophage infections, glucose starvation-dependent inflammasome activation requires some initial fungal escape, which then leads to extracellular replication and reduced glucose concentration. In animal infections, the formation of a fungal mass in tissues and organs likely leads to a low glucose microenvironment. These fungal masses are very similar to solid tumors that create low glucose environments [48, 49], and *C*. *albicans* infection lowers glucose levels in the microenvironment of the kidney [39]. We have shown that activation of macrophages by the fungal cell wall component ß-glucan in combination with interferon-γ is sufficient to cause them to depend on glucose for survival [39]. As further discussed below, our data shows that inflammasome activation by glucose starvation is linked to macrophage viability. Therefore, inflammasome activation in low-glucose environments could occur following recognition of ß-glucan regardless of phagocytosis of *C*. *albicans* by macrophages, meaning that sensing of infection is mediated by the levels of glucose needed to support macrophage viability. Our study used primary mouse BMDMs, and it will be important to understand how tissue macrophage respond to glucose levels. Future work should also determine how macrophage glucose metabolism dictates inflammasome activation and disease outcomes *in vivo*. We show that *C*. *albicans* strain P78042, which does not form robust hyphae and only triggers glucose starvation-dependent inflammasome activation in macrophages, can nevertheless induce production of IL-1β in the murine infection model. This result suggests that the glucose starvation-dependent mechanisms of inflammasome activation might be operational not only *ex vivo* in macrophages, but also *in vivo* in animal infections.

As mentioned, the glucose starvation-dependent mechanism of inflammasome activation by *C*. *albicans* that we describe here is distinct to the previously described mechanism driven by fungal hyphae. Several lines of evidence support this conclusion. First, there is temporal separation–the hyphae-dependent mechanism occurs early after infection (during Phase I activation), while the glucose-dependent mechanism occurs only when fungal load is sufficiently high to cause glucose starvation in macrophages (during Phase II activation). The temporal separation of these two mechanisms is clearly seen in infections with a *Candida* mutant that consumes glucose more slowly. The *tye7Δ/Δ gal4Δ/Δ* mutant makes hyphae and triggers Phase I inflammasome activation normally, but Phase II activation is delayed. Second, the glucose starvation-dependent mechanism of inflammasome activation is seen with *C*. *albicans* strains that are very poorly hyphal or even unable to form hyphae altogether. The independence from hyphal formation is also shown by the fact that increasing glucose levels reduces inflammasome activation (Figs 6 and 7), although it triggers more potent hyphal growth by *Candida* [39]. Third, the hyphae-driven Phase I mechanism leads to pyroptotic macrophage death, but Phase II activation of the inflammasome by glucose starvation does not. This is seen in experiments with the NLRP3 inhibitor MCC950, which reduces the glucose starvation-dependent burst of ASC speck formation, but it does not rescue macrophages from cell death (Fig 4B). Although inflammasome activation by glucose starvation does not lead to pyroptosis, it does lead to the other key functional output of activated inflammasome—production of the pro-inflammatory cytokine IL-1β, showing functional relevance.

Interestingly, our analysis of a compendium of diverse *C*. *albicans* isolates showed that the Phase I, hyphae-dependent wave of inflammasome activation is seen only in exceptional experimental conditions–only with a subset of clinical strains, and only when the infection load of hyphae is high (MOI of 6), but not when it is low (MOI of 1) (Fig 3G). This means that detection of hyphae *per se* is not sufficient to activate the inflammasome, the load of hyphae needs to be sufficient. How fungal hyphae are recognised by macrophages to activate the inflammasome is not completely clear, but mechanisms such as rupture of phagosomal membrane by hyphal growth, production of the toxin candidalysin and recognition of cell surface components have been proposed [2, 25–29, 31, 32, 42]. It is easy to imagine that any or all of these mechanisms could depend on hyphal load being sufficiently high. In contrast, Phase II, glucose starvation-induced inflammasome activation is seen in all conditions that we tested and is dominant across phylogenetically and phenotypically diverse fungal isolates. Our analysis further showed that only a subset of *C*. *albicans* clinical isolates trigger robust Phase I macrophage death that depends significantly on pyroptosis (S3 Fig and S4 Fig). This suggests that the broad relevance of macrophage pyroptosis in fungal infections remains to be fully understood. Based on these considerations we conclude that, although hyphae-dependent mechanisms play a role under some conditions, the dominant way by which *C*. *albicans* activates the inflammasome is by starving macrophages of glucose.

What is the mechanism of glucose starvation-dependent activation of NLRP3 by *C*. *albicans*? ROS have been suggested to activate NLRP3 [44–46], but reduction of ROS levels by the antioxidant NAC did not reduce ASC speck formation in our experiments. In *Candida*-infected macrophages glycolysis is the only source of ATP [39]. Glucose starvation caused by *C*. *albicans* will cause a drop in intracellular ATP levels in macrophages, which could contribute to NLRP3 activation [50]. Furthermore, our results show that, in macrophage populations, NLRP3 activation by *Candida*-induced glucose starvation occurs concurrently with hyperpolarization of mitochondria and macrophage cell death. The major job of the NLRP3 pathway is to detect dangers to membrane integrity and cellular viability and to trigger alarm *via* IL-1β secretion [22]. Mitochondria are central to cellular viability and metabolic homeostasis, and, as such, mitochondrial dysfunction has been implicated in NLRP3 inflammasome activation by various mechanisms, reviewed in [51]. While in our imaging experiments staining of macrophage nuclei by the dye Draq7 occurred after ASC speck formation, loss of plasma membrane integrity must precede influx of Draq7 into cells and our imaging does not report on the exact timing of the initial damage to the membrane. Loss of membrane integrity leads to potassium efflux, which is the common up-stream event leading to NLRP3 activation in response to a variety of inflammasome activators [22]. Inhibiting potassium efflux repressed glucose starvation-dependent inflammasome activation by *C*. *albicans* and also reduced mitochondrial hyperpolarisation (Fig 4). Collectively, our data fit with NLRP3 being activated by damage to mitochondrial function and cellular homeostasis and the loss of membrane integrity upon *Candida*-induced glucose starvation.

Our data seems to contrast with previous work showing that metabolic reprogramming and glycolysis are needed to support production of IL-1β in response to fungal and bacterial challenge [36, 52]. There are several reasons that could explain these contrasting conclusions. In our experiments, macrophages were challenged with *C*. *albicans* in the presence of glucose (even the medium without additional glucose contains a small amount of glucose due to serum). Therefore, macrophages were sensing *C*. *albicans* and switching on their glycolytic metabolism in a condition where glycolysis could be performed, and their glycolytic metabolism was perturbed only later in infection due to glucose depletion by robust fungal replication. This scenario is likely to reflect the situation *in vivo*, where macrophages need to detect an increase in the microbial load over time. In the previous study, monocytes were sensing *C*. *albicans* in a condition where glycolysis is not possible due to pre-treatment with the glycolysis inhibitor 2-deoxyglucose [52]. This could produce a physiological and metabolic state that incompatible with cytokine production. Also, transcription of IL-1β by HIF1α was shown to require glycolysis [36], while we measured proteolytic processing of the IL-1β protein and its subsequent secretion in *C*. *albicans*-infected macrophages. It is possible that different stages of cytokine production (gene transcription, protein maturation) require distinct metabolic inputs. A further intriguing observation is that, in contrast to *C*. *albicans*, when macrophages are infected with the intracellular bacterium *Salmonella typhimurium* impairment of host glycolysis leads to pyroptosis [53]. This shows that the immunometabolism-inflammasome axis can lead to distinct functional outcomes, possibly reflecting intracellular events in inflammasome activation (in the case of *Salmonella*), as opposed to macrophages sensing a nutritional change in the infection niche (in the case of *Candida*).

Modulation of immunometabolism and glucose homeostasis has promise in treating infections [35], and our study now suggests that maintaining host glucose homeostasis could modulate inflammatory pathology in fungal infections. This is supported by our data showing that supplementation of glucose reduced inflammasome activation and IL-1β production in response to *C*. *albicans*. How these metabolic approaches to infection control could be combined with strategies targeting NLRP3 [43] is an exciting question for future studies.

## Materials and methods

### Ethics statement

Animal experiments were approved by the Monash University Animal Ethics Committee (approval number MARP-2015-170/ID 14292). Human blood collection and experiments were approved by the Monash University Human Ethics Committee (project ID: 21685).

### Strains and culture conditions

The *C*. *albicans* strains used in this study are listed in S1 Table. All clinical strains of *C*. *albicans* except for SC5314 were obtained from BEI Resources, NIAID, NIH (www.beiresources.gov). The *tye7Δ/Δ gal4Δ/Δ* mutant and the relevant control strain were a gift from Malcolm Whiteway [47]. Media culture was YPD agar plates (1% yeast extract, 2% peptone, 2% glucose, and 2% agar). Single colonies of *C*. *albicans* (various strains) were patched on a YPD plate and the plate was incubated at 30°C for 12 h. Before infection of macrophages, fungal cells were resuspended in PBS and counted in a hemocytometer.

### Hyphal Index calculation

For each of the 21 clinical isolates of *C*. *albicans*, yeast cells were counted in a hemocytometer and added to 24-well tissue culture wells (at 3x10^6^ cells / well) containing 750 μl tissue culture media lacking macrophages (to assess liquid hyphal formation), or in the same media containing 5x10^5^ murine bone marrow-derived macrophages / well (to assess macrophage hyphal formation). The media was RPMI 1640 supplemented with 12.5 mM HEPES, 15% FBS, 20% L-cell conditioned medium (containing macrophage colony-stimulating factor), and 100 units/mL penicillin-streptomycin. For liquid hyphal formation, filamentation was allowed to proceed for 3 h at 37°C + 5% CO_2_, and then multiple photographs were taken using DIC microscopy. For macrophage hyphal formation, cells were allowed to co-incubate for 1 h to allow for phagocytosis, and then non-phagocytosed fungal cells were removed by washing three times with PBS. Infected macrophages were incubated for 3 h at 37°C + 5% CO_2_ and then multiple photographs were taken using DIC microscopy.

To calculate hyphal index, 200 cells (chosen as a random representation from multiple images) were measured for length and width in μm using ImageJ software’s Measurement tool for segmented line. Length was defined as the distance from mother cell to tip of hyphae (for hyphae with multiple branches, the longest branch was measured), and width was defined as the widest distance measurement within 5 μm proximity of the hyphal tip (or the widest distance across the yeast cell if hyphal formation did not occur). For each cell, hyphal index was calculated as length divided by width, and the hyphal index values for 200 cells were averaged for each isolate. This index is therefore indicative of how long and thin the hyphae are (indicating true hyphae), versus shorter and broader (indicating so-called pseudohyphal forms and budding yeast cells). Finally, these scores were normalized to a scale of 0–5, with benchmark strain SC5314 (which showed uniform hyphae formation in liquid media and in macrophages) being assigned a score of 5.

### Isolation of murine bone marrow derived macrophages (BMDMs)

Murine BMDMs isolation and differentiation was performed as previously described [39, 42]. Briefly, murine monocytes were extracted from the femur and tibia bones of C57BL/6 wild type or inflammasome mutant mice (*Casp1/11*^*-/-*^, *Nlrp3*^*-/-*^ and *Gsdmd*^*-/-*^) (6 to 8 weeks of age) and differentiated in RPMI 1640 media supplemented with 20% L-cell conditioned media, 15% fetal bovine serum (FBS; Serana, Fisher Biotech), 100 U/L penicillin-streptomycin (Sigma-Aldrich) and 12.5 mM HEPES. Following extraction, monocytes were incubated at 37°C and 5% CO_2_ for 24 h. Non-adherent monocytes were then collected and incubated at 37°C and 5% CO_2_ for a further seven days before seeding.

### Isolation of human monocyte-derived macrophages (hMDMs)

Human monocytes were isolated from blood collected from healthy donors. Following Histopaque-1077 (Sigma-Aldrich) density gradient centrifugation, CD14 and CD16 positive cells were enriched from isolated monocytes using the Pan Monocyte Cell Isolation (Miltenyi Biotech) magnetic labelling system. Enriched monocytes were then differentiated into human peripheral blood mononuclear cells by incubation in RPMI 1640 media supplemented with 15 mM HEPES, 10% FBS, 100 U/L penicillin-streptomycin and 50 ng/mL macrophage colony-stimulating factor (M-CSF, R&D Systems) at 37°C and 5% CO_2_ for seven days.

To detach differentiated hMDMs, culture media was replaced with PBS supplemented with 2 mM EDTA and 2% FBS and incubated at 37°C and 5% CO_2_ for 10 minutes. hMDM culture flasks were then repeatedly rinsed with PBS supplemented with 2mM EDTA and 2% FBS using a 10 mL syringe and 18G needle until complete detachment of cells were seen. These cells were then seeded at 5 x 10^4^ cells/well in 96-well plates and incubated in RPMI 1640 media supplemented with 15 mM HEPES, 10% FBS, 100 U/L penicillin-streptomycin at 37°C and 5% CO_2_ overnight.

### Live cell imaging assays

For murine BMDMs, live cell imaging experiments were performed as previously described [39, 42]. Briefly, BMDMs were seeded in tissue culture-treated plates as follows: 5x10^5^ cells / well were used in 24-well plates and 1x10^5^ cells / well were used in 96-well plates. CellTracker^TM^ Green CMFDA dye (Thermo Fisher C7025) was used at 1 μM final concentration, and staining performed for 20 min in serum-free RPMI 1640 medium. To initiate infection, macrophages were co-incubated with *C*. *albicans* for 1 h (MOI of 6:1 or 1:1 *Candida*:macrophage), then washed with PBS (x3) and stained with 0.6 μM DRAQ7 (Abcam) for detection of dead cells and 50 nM tetramethylrhodamine, methyl ester (TMRM; Thermo Fisher T668) for monitoring macrophage mitochondrial membrane potential. The media volume at the start of live cell imaging was 750 μl for 24-well format and 150 μl for 96-well format. Live cell imaging was performed on a Leica AF6000 LX epifluorescence microscope. The microscopy images and data were collected and analysed as described previously [39, 42].

The immortalised mCerulean-tagged ASC inflammasome reporter macrophages were obtained from Eicke Latz [41]. Culturing was done in RPMI containing 12.5 mM HEPES and 10% FBS, at 1x10^5^ cells/well in 96-well tissue culture plates. Infection with *C*. *albicans* was performed at MOI of 6:1 or 1:1 depending on the experiment (as indicated in the Figure legends). After the initial challenge and phagocytosis, live cell imaging was set up as described previously [42]. For data analysis, a custom-made journal suite in MetaMorph was used to count the number of macrophages with diffuse cyan, cyan ASC specks and dead cells as determined by staining positive with DRAQ7. The total number of macrophages was obtained by adding together the cells with diffuse cyan staining and those containing a cyan ASC speck.

For human MDMs (hMDMs), live cell imaging was performed as described above for BMDMs with the following modifications: seeded hMDMs were first exposed to 1μM CellTracker^TM^ Green CMFDA dye in serum-free RPMI 1640 media at 37°C and 5% CO_2_ for 30 mins. This serum-free media was then replaced with in RPMI 1640 media supplemented with 15 mM HEPES, 10% FBS and 50 ng/ml lipopolysaccharide (LPS; Sigma) and incubated at 37°C and 5% CO_2_ for 2 h. hMDMs were then challenged with *C*. *albicans* strains at MOI of 6:1 (*Candida*:macrophage) for 1 h, and the remaining steps for live cell imaging were performed as described above.

### Doubling time and XTT measurements

*C*. *albicans* clinical isolates were grown overnight in YPD at 30°C. The following day, cells were washed once in sterile water and then diluted to a final concentration of 0.1 OD_600_ in 200 μl minimal media (6.7 g/L Yeast Nitrogen Base with ammonium sulphate, supplemented with either 2% glucose or 2% glycerol and amino acids) and then transferred to a clear flat bottom 96-well plate. Plates were continuously shaken and OD_600_ readings recorded every 15 minutes for 24 hours, at 37°C temperature using a Tecan Spark 10M microplate reader. Doubling times from the growth curves were calculated using the formula T_d_ = (t_2_-t_1_)* log(2) / (log (Q_2_/Q_1_)) where t_1_ and t_2_ are at the beginning and end of exponential phase, respectively, and Q represents the OD_600_ reading at the corresponding time. Data are averages of three biological replicates per condition (with 3 technical replicates performed per biological replicate).

For measuring metabolic activity by XTT, selected clinical isolates were grown overnight at 30°C in minimal media supplemented with 2% glucose. The next day, cells were diluted to a final OD_600_ = 0.2 in minimal media supplemented with glucose and grown for 4 h at 37°C. Then 100 μl cultures (in triplicates) of each clinical isolate were transferred to a 96-well plate, centrifuged and supernatants removed. To each well, 200 μl of XTT/menadione solution (freshly prepared by mixing 1 μl of the 10 mM of menadione with 10 mL of 0.5 mg/ml XTT solution) was added and the plate incubated in the dark for 2 h at 37°C for colour development. Then 100 μl of the resulting coloured supernatants from each well were transferred into a new microtiter plate and absorbance at 490 nm was measured using Tecan Spark 10M microplate reader.

### Drug and media treatments

For select live cell microscopy experiments, 10 μM MCC950, 10 μM MCC6642, 10 μM CA-074-Me, 30 mM KCl and DMSO for controls where needed were included in the tissue culture media. Cells were pre-treated for 1 h, 2 h (hMDMs) or 3 h (inflammasome mutant BMDMs) prior to *Candida* infection. This was followed by challenge with *Candida*, and the same concentrations were again added to media afterwards.

In order to perform the experiments in differing concentrations of glucose, or with the addition of galactose, sorbitol or mannitol, the base medium was RPMI 1640 medium lacking glucose (Thermo Fisher 11879020) with addition of 10% FBS. We have previously determined that this medium, designated “no glucose addition”, contains an approximate concentration of glucose at 0.65 mM [39]. Various carbon sources were added to this medium at the concentrations indicated in the Figure legends. This media was added to macrophages post-phagocytosis (after the 1 h *Candida*:macrophage co-incubation and 3 x PBS washes).

### Intracellular ROS measurement

BMDMs (1.25x10^5^) were seeded into black clear-bottom 96-well plates and incubated overnight at 37°C + 5% CO_2_. Then, cells were loaded in the dark with 20 μM Carboxy-H_2_DCFDA (ThermoFisher C400) in pre-warmed PBS for 30 min at 37°C + 5% CO_2_ and washed once with pre-warmed PBS. Macrophages were then infected with *C*. *albicans* (SC5314 at 6:1 and 1:1 MOI) in the presence or absence of 5 mM ROS scavenger N-acetyl-L-cysteine (NAC; pH adjusted to 7.2), and fluorescence readings (excitation 492 nm / emission 535 nm) were recorded using a Tecan Spark 10M microplate reader immediately after infection and at 10 minute time points over the course of 24 h. Values were subtracted from initial reading to measure fluorescence accumulation over the course of infection. All conditions were measured in triplicate and from 2 independently set up experiments.

### Glucose measurements

ASC-mCerulean-expressing immortalized macrophages were infected with *Candida* strains SC5314 (MOI 6:1), P78042 (MOI 6:1), or left untreated. Every 3 h post-phagocytosis and over the course of 24 h, tissue culture supernatants were collected and glucose concentrations measured using the Amplex^TM^ Red Glucose/Glucose Oxidase Assay Kit (Thermo Fisher A22189) as per manufacturer’s instructions. The Tecan Spark 10M microplate reader was used to measure absorbance at 560 nm (in triplicates).

### Western blots and ELISA

For assaying IL-1β BMDMs were primed with 50 ng/ml LPS (ultra-pure from Sigma) for 3 h. Primed BMDMs were challenged with *C*. *albicans* at 6:1 MOI. The experiment was performed in 24-well tissue culture-treated plates and the tissue culture media contained 0, 10, 40, or 100 mM added glucose to RPMI 1640 lacking glucose. After the phagocytosis step, the volume of media was reduced from 750 μl to 250 μl to increase the concentration of IL-1β and improve detection. At 3 h post-phagocytosis supernatants and lysates were collected for analysis by Western blot or ELISA. As a positive control for inflammasome activation and IL-1β processing, BMDMs were treated with 10 μM nigericin (Thermo Fisher).

Western blot experiments were performed using 10 μl of supernatant or 5 μl of lysate separated on 15% SDS-PAGE followed by transfer to 0.45 μm nitrocellulose membrane. Blocking and antibody incubations were in TBS with 5% skim milk and 0.1% Tween 20 (TBS-T) (1 h, room temperature). Membranes were incubated overnight with 1:1000 dilution of mouse IL-1β primary antibody (R&D Systems; AF-401-NA), washed in TBS-T and then incubated with anti-goat HRP secondary antibody at 1:10000 dilution for 1 hour. Antibodies were diluted in TBS-T + 5% skim milk. Detection was performed using luminol-based enhanced chemiluminescence (SuperSignal^TM^ West Dura Extended Duration Substrate; Thermo Fisher 34075). After IL-1β detection in lysates, membranes were stripped and re-probed using anti-actin mouse monoclonal antibody (Millipore; MAB1501) diluted at 1:5000, and then anti-mouse IgG secondary antibody (Sigma; A4416) at 1:20000 dilution. Lysate membranes were also stained with Ponceau S for total protein detection. Uncropped images of Western blots are shown in S5 Fig and S7 Fig.

The levels of released IL-1β (in supernatants) were measured using the mouse IL-1β ELISA kit (R&D Systems; DY401), following manufacturer’s instructions. The time point was 3 h post-phagocytosis.

### Mouse infection experiments

Female BALBc mice (6–8 weeks old) were infected with 1x10^6^ CFUs of *C*. *albicans* SC5314 or P78042 intravenously (tail vein injection). Kidneys were collected at 24 h post infection, homogenized in PBS with protease inhibitor cocktail (Roche), and then centrifuged. Supernatants were snap frozen on dry ice and stored at -80°C. IL-1β levels in kidney homogenates were determined using the mouse IL-1β ELISA kit (R&D Systems; DY401) as above.

### Statistical analysis

Prism 7 (GraphPad) software was used to perform statistical analysis and *p*-value of <0.05 considered significant. Predominantly, two groups were compared using the unpaired, two-tailed Student’s *t* test. Statistical analysis of correlation plots was calculated using Pearson’s correlation coefficient, and statistical analysis of ELISA experiments and ASC speck measurements with multiple groups were calculated using one-way ANOVA and Tukey’s multiple comparison test. Figure legends describe the number of biological and technical replicates and statistical values where relevant. The numerical values used for constructing graphical data displayed in the figures are shown in S1 Data.

## Supporting information

S1 Fig Correlation of hyphal morphogenesis of *C. albicans* strains *in vitro* and in macrophage infections. (A) Side-by-side comparison of microscopy images of *C*. *albicans* clinical isolates 3 h after addition to liquid tissue culture media (labeled as “*Candida*”) compared to *Candida* infecting macrophages (labeled as “*Candida* + macrophages”). For each isolate, 200 hyphae were measured. Values in top-left corner of each image indicate the hyphal index calculation, as described in the Methods. Scale bar is 20 μm. The images of *Candida* infecting macrophages are the same as in Fig 1B. (B) Correlation plot of hyphal index in macrophages versus hyphal index in liquid tissue culture media, with the Pearson’s correlation coefficient (*r*) and *p* values as indicated. (C) Correlation plot of hyphal index in liquid tissue culture media with the doubling time of each clinical isolate grown at 37°C in minimal media with glucose as the carbon source, with the Pearson’s correlation coefficient (*r*) and *p* values as indicated. Our data for the doubling times for these clinical isolates in minimal medium at 37°C correlates with the data of [40] obtained from growth curves in rich medium (YPD) at 37°C (Pearson’s correlation coefficient value of 0.7281, *p* value of 0.0002). (D) Correlation plot of hyphal index in liquid tissue culture media with the doubling time of each clinical isolate grown at 37°C in minimal media with glycerol as the carbon source, with the Pearson’s correlation coefficient (*r*) and *p* values as indicated. (E) Correlation plot of XTT measurements with the doubling time (using same data as in C) at 37°C in minimal media with glucose as the carbon source with the Pearson’s correlation coefficient (*r*) and *p* values as indicated. XTT assay was done from indicated clinical strains grown for 4 h in minimal media with glucose as the carbon source at 37°C. Data are the mean values and SEM from 4 independent experiments. (F) Correlation plot of hyphal index in macrophages with the doubling time of each clinical isolate grown at 37°C in minimal media with glucose as the carbon source, with the Pearson’s correlation coefficient (*r*) and *p* values as indicated. (G) Correlation plot of hyphal index in macrophages with the doubling time of each clinical isolate grown at 37°C in minimal media with glycerol as the carbon source, with the Pearson’s correlation coefficient (*r*) and *p* values as indicated.
(TIF)

S2 Fig Comparison of *C. albicans* strains in relation to infection rate, growth rate, hyphal formation and macrophage killing. (A) Microscopy images of *Candida* (stained with calcofluor white) following macrophage phagocytosis. Murine BMDMs were infected with the indicated *C*. *albicans* strains at 2:1 MOI. One hour post-phagocytosis, the macrophages were permeabilised using 0.1% Triton-X 100 and then stained with 10 μg/ml calcofluor white. At least 400 macrophages were counted for each strain and phagocytic index is displayed in the table. (B) Same experiment as in S2A Fig, but the macrophages were infected at 1:1 MOI (note that the other clinical isolates also showed a comparable phagocytosis efficiency in macrophages, based on live cell imaging observations). (C) Correlation plots comparing start of Phase II macrophage death vs. doubling time, and hours to 50% macrophage death vs. doubling time (doubling time of each clinical isolate was measured at 37°C in minimal media). Due to low macrophage killing during the course of the experiment, P94015 is not included. For each graph, the Pearson’s correlation coefficient (*r*) and *p* values are indicated.
(TIF)

S3 Fig Macrophage killing kinetics of a diverse compendium of *C. albicans* clinical strains. Primary murine BMDMs were infected with each *C*. *albicans* isolate at 6:1 MOI and assessed for cell death by live cell imaging over 24 hours. Each graph compares a clinical isolate with the benchmark strain SC5314 and uninfected macrophages control, all assayed in the same live cell imaging experiments. Note that the following isolates were assayed in the same experiments and therefore are being compared with the same control data for strain SC5314 and uninfected macrophages: P60002, GC75, P75063 and P75016; L26, 19F, P37005 and P37039; P76067, P76055 and P57072; P34048, P57055 and P78042. For the remaining strains (P87, P37037, P78048, 12C, P75010, P94015), they were assayed with a different set of strains in the two experiments; therefore, they share control data with some strains in the first experiments, and with other strains in the second. Accordingly, the control data displays differently. Each clinical isolate is displayed in a separate graph for clarity in presentation and ease of comparisons to the prototype strain SC5314. Data are the mean values and SEM from 2 independent experiments involving 4 different colonies of each *Candida* strain (each colony was treated as a biological replicate) and at least 2000 macrophages surveyed for each strain, per experiment. The data for strains P87, L26, P78048, P57055, P78042 and P75010 shown here is the same as the data shown in Fig 1C. Here we show it again to have a complete set of *C*. *albicans* clinical strains for comparison.
(TIF)

S4 Fig All clinical strains of *C. albicans* trigger inflammasome activation, with two temporally distinct events. Macrophages were infected with the indicated *C*. *albicans* strains at 6:1 MOI or treated with nigericin (10 μM). Displayed in each graph are % dead macrophages, % macrophages containing an ASC speck, and % macrophages with hyperpolarized mitochondria as quantified in live cell imaging experiments. Data are the mean values and SEM from 3 independent experiments. The data for SC5314 and P78042 is the same as shown in Fig 3. Here we show it again to have a complete set of clinical isolates for comparison. The y-axis is set to 50% maximum to show clearly the ASC speck formation events, but macrophage cell death continued accumulating and eventually reached 100% for all *C*. *albicans* strains. We note that with the ASC-mCerulean expressing immortalised BMDMs the kinetics of macrophage cell death in response to *C*. *albicans* clinical strains was generally faster compared to experiments with primary BMDMs. Nevertheless, as in primary BMDMs, in the ASC-mCerulean immortalised cells the *C*. *albicans* strains that form robust hyphae triggered macrophage cell death more rapidly than those with less robust hyphal formation, meaning that the relationship between morphogenesis and macrophage cell death was preserved.
(TIF)

S5 Fig Entire immunoblots for detection of IL-1β from strains SC5314 and P78042. Murine bone marrow-derived macrophages were primed with LPS (50 ng/ml) for 3 h, followed by challenge with *Candida* strains SC5314 or P78042 (MOI 6:1), nigericin (10 μM), or uninfected. At 12 h post-challenge, supernatants and lysates were collected and analysed by immunoblot for IL-1β. Bottom image contains Ponceau S staining of total protein from lysates. Displayed in the boxes are the lanes that were spliced together to construct Fig 3E.
(TIF)

S6 Fig Inactivation of the inflammasome pathway does not block Phase II macrophage death in infections with diverse *C. albicans* clinical isolates. Wild type, *Casp1/11*^*-/-*^, *Nlrp3*^*-/-*^ and *Gsdmd*^*-/-*^ murine bone marrow-derived macrophages were primed with LPS (50 ng/ml) for 3 h followed by infection with *C*. *albicans* strains SC5314, L26 and P57055 at 6:1 MOI. Experiment 1 and 2 were performed on two different days, using the same preparations of murine BMDMs from each inflammasome mutant (one mouse per strain) and independent cell cultures of each *C*. *albicans* clinical isolate. In each of the experiments, all data were collected together, but each *C*. *albicans* isolate is displayed in a separate graph for clarity. The uninfected controls are the same in the three graphs from the same experiment. Shown are the average and SEM of 3 technical repeats for *Candida* infections and two technical repeats for uninfected controls. At least 3000 macrophages were counted for each of the samples.
(TIF)

S7 Fig Entire immunoblots for detection of IL-1β and actin, from three independent experiments. Murine bone marrow-derived macrophages (harvested independently from three different mice) were primed with LPS (50 ng/ml) for 3 h, followed by treatment with *C*. *albicans* hyphal strain SC5314 (MOI 6:1), nigericin as positive control (10 μM), or uninfected as negative control. The starting tissue culture media (~0.65 mM glucose) contained 0, 10, 40, or 100 mM added glucose. At 3 h post-infection, supernatants and lysates were collected and analysed by immunoblot. For each experiment, bottom image contains Ponceau S staining of total protein from lysates.
(TIF)

S8 Fig The effect of glucose on inflammasome activation is specific, not due to changes in osmolarity and not recapitulated by other carbon sources. The conditions in panels A-D were all assayed in the same live cell imaging experiments; therefore, the data for control conditions (with no carbon source addition) are the same between different panels. For clarity in presentation, the data are displayed in separate graphs. (A) ASC-mCerulean-expressing immortalized macrophages following infection with *Candida* hyphal strain SC5314 (MOI 6:1) with the tissue culture media (~0.65 mM glucose) containing 0, 10, or 40 mM added glucose. Shown are % macrophages containing an ASC speck, % macrophages with hyperpolarized mitochondria, and % dead macrophages, as quantified in live cell imaging experiments. Data are the mean values and SEM from 2 independent experiments involving at least 9000 macrophages surveyed for each condition, per experiment. (B) Same as panel A, but with the tissue culture media containing 0, 10, or 40 mM added galactose (note that the “no galactose addition” controls are the same data as the “no glucose addition” controls in panel A). (C) Same as panel A, but with the tissue culture media containing 0, 10, or 40 mM added sorbitol (note that the “no sorbitol addition” controls are the same data as the “no glucose addition” controls in panel A). (D) Same as panel A, but with the tissue culture media containing 0, 10, or 40 mM added mannitol (note that the “no mannitol addition” controls are the same data as the “no glucose addition” controls in panel A).
(TIF)

S1 Movie The yeast-locked clinical isolate P94015 can kill and escape from macrophages in low frequency bursting events. Murine bone marrow-derived macrophages were infected with P94015 at 6:1 MOI and imaged by live cell microscopy for 36 h. At ~10 h post infection, P94015 cells can be seen bursting and escaping from a recently killed macrophage (see bottom right corner of the movie). Then, external yeast cells start appearing in larger numbers, eventually leading to rapid accumulation of macrophage death after 24 h. Images are a merging of bright field (gray) and DRAQ7 (far-red) channels. Video contains 145 time points imaged every 15 minutes over the course of 36 h, compressed into 14 images / second).
(AVI)

S1 Table Strains used in this study. (DOCX)

S1 Data The underlying numerical values for the graphical data displayed in the figures. Each sheet contains data for the indicated figures and figure panels.
(XLSX)
