# Supplementary figures and images for: Metabolic competition between host and pathogen dictates inflammasome responses to fungal infection
Source: PLoS Pathog. 2020 Aug 4;16(8):e1008695. doi: 10.1371/journal.ppat.1008695 (PMC7433900; doi:10.1371/journal.ppat.1008695)

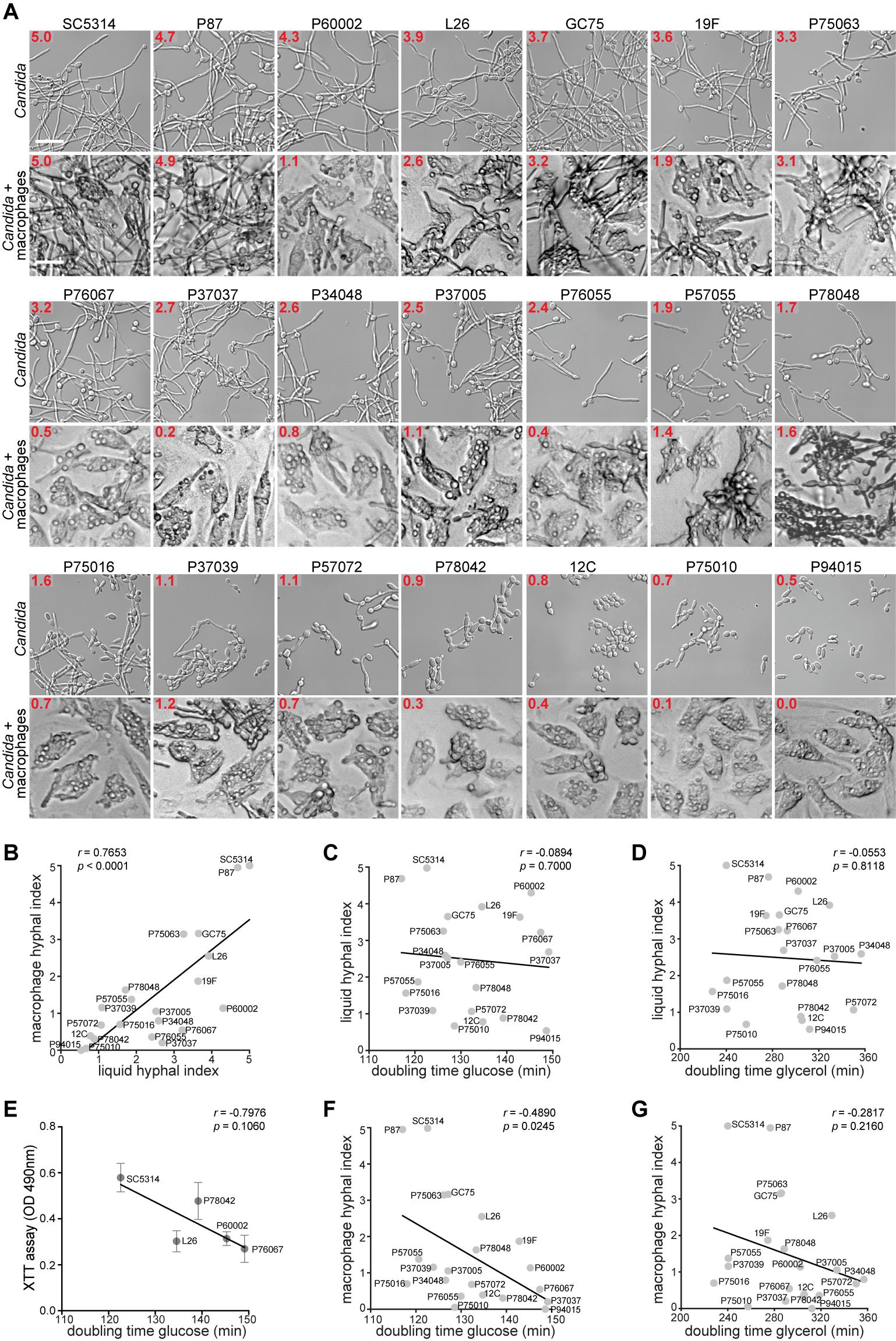

Supplement: S1 Fig — (A) Side-by-side comparison of microscopy images of C. albicans clinical isolates 3 h after addition to liquid tissue culture media (labeled as “Candida”) compared to Candida infecting macrophages (labeled as “Candida + macrophages”). For each isolate, 200 hyphae were measured. Values in top-left corner of each image indicate the hyphal index calculation, as described in the Methods. Scale bar is 20 μm. The images of Candida infecting macrophages are the same as in Fig 1B. (B) Correlation plot of hyphal index in macrophages versus hyphal index in liquid tissue culture media, with the Pearson’s correlation coefficient (r) and p values as indicated. (C) Correlation plot of hyphal index in liquid tissue culture media with the doubling time of each clinical isolate grown at 37°C in minimal media with glucose as the carbon source, with the Pearson’s correlation coefficient (r) and p values as indicated. Our data for the doubling times for these clinical isolates in minimal medium at 37°C correlates with the data of [40] obtained from growth curves in rich medium (YPD) at 37°C (Pearson’s correlation coefficient value of 0.7281, p value of 0.0002). (D) Correlation plot of hyphal index in liquid tissue culture media with the doubling time of each clinical isolate grown at 37°C in minimal media with glycerol as the carbon source, with the Pearson’s correlation coefficient (r) and p values as indicated. (E) Correlation plot of XTT measurements with the doubling time (using same data as in C) at 37°C in minimal media with glucose as the carbon source with the Pearson’s correlation coefficient (r) and p values as indicated. XTT assay was done from indicated clinical strains grown for 4 h in minimal media with glucose as the carbon source at 37°C. Data are the mean values and SEM from 4 independent experiments. (F) Correlation plot of hyphal index in macrophages with the doubling time of each clinical isolate grown at 37°C in minimal media with glucose as the carbon source, with [file ppat.1008695.s001.tif]

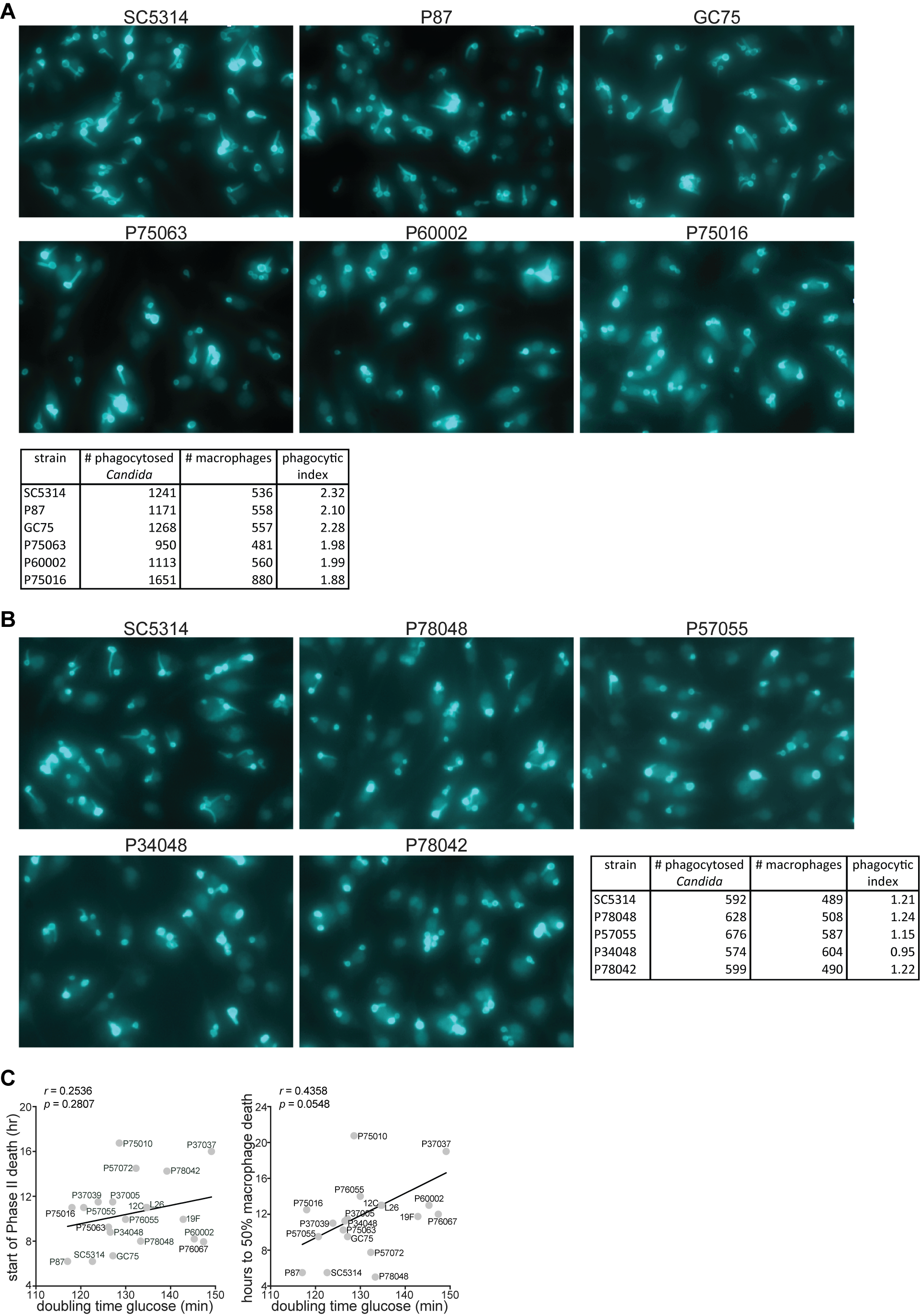

Supplement: S2 Fig — (A) Microscopy images of Candida (stained with calcofluor white) following macrophage phagocytosis. Murine BMDMs were infected with the indicated C. albicans strains at 2:1 MOI. One hour post-phagocytosis, the macrophages were permeabilised using 0.1% Triton-X 100 and then stained with 10 μg/ml calcofluor white. At least 400 macrophages were counted for each strain and phagocytic index is displayed in the table. (B) Same experiment as in S2A Fig, but the macrophages were infected at 1:1 MOI (note that the other clinical isolates also showed a comparable phagocytosis efficiency in macrophages, based on live cell imaging observations). (C) Correlation plots comparing start of Phase II macrophage death vs. doubling time, and hours to 50% macrophage death vs. doubling time (doubling time of each clinical isolate was measured at 37°C in minimal media). Due to low macrophage killing during the course of the experiment, P94015 is not included. For each graph, the Pearson’s correlation coefficient (r) and p values are indicated. (TIF) [file ppat.1008695.s002.tif]

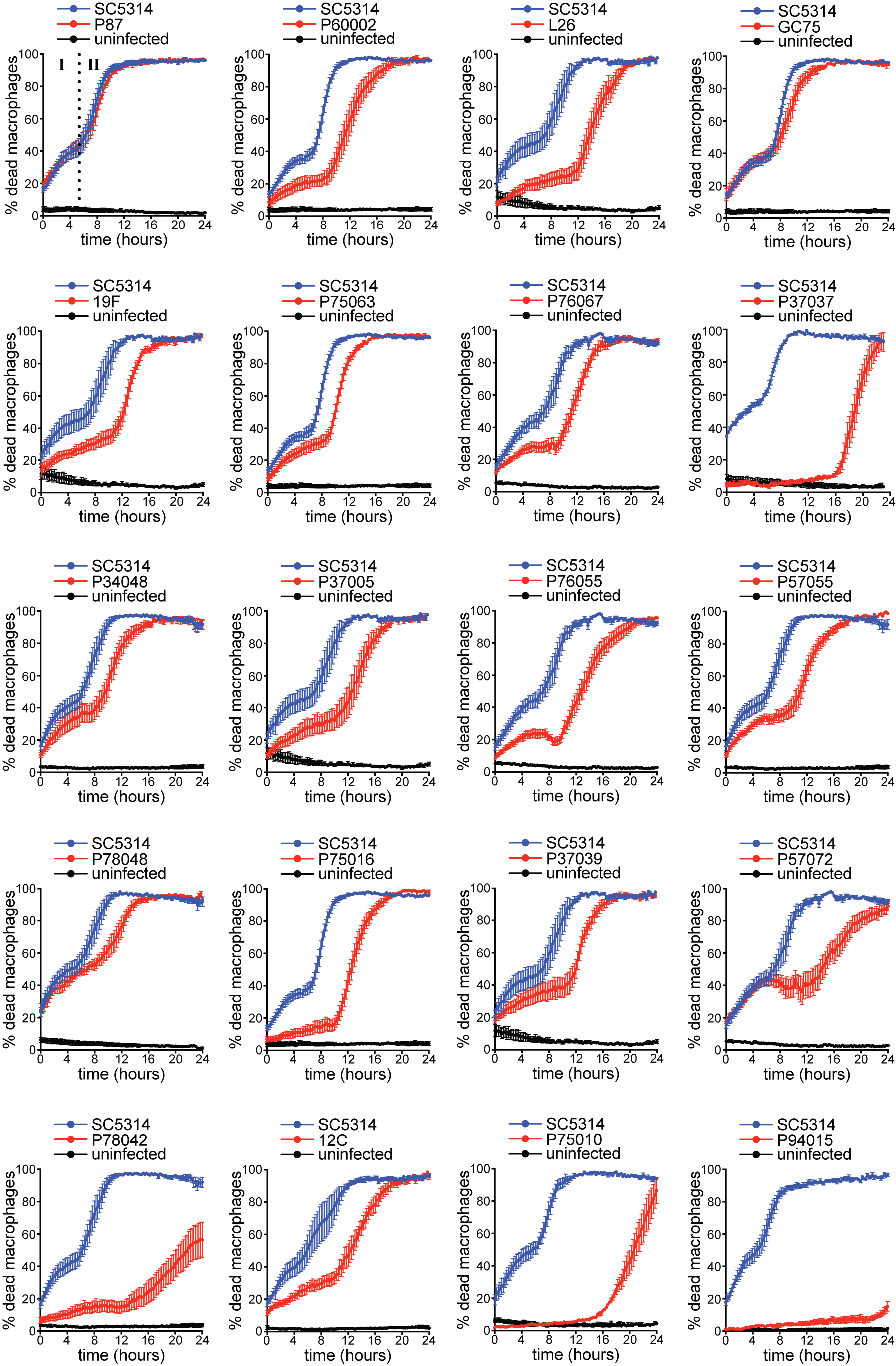

Supplement: S3 Fig — Primary murine BMDMs were infected with each C. albicans isolate at 6:1 MOI and assessed for cell death by live cell imaging over 24 hours. Each graph compares a clinical isolate with the benchmark strain SC5314 and uninfected macrophages control, all assayed in the same live cell imaging experiments. Note that the following isolates were assayed in the same experiments and therefore are being compared with the same control data for strain SC5314 and uninfected macrophages: P60002, GC75, P75063 and P75016; L26, 19F, P37005 and P37039; P76067, P76055 and P57072; P34048, P57055 and P78042. For the remaining strains (P87, P37037, P78048, 12C, P75010, P94015), they were assayed with a different set of strains in the two experiments; therefore, they share control data with some strains in the first experiments, and with other strains in the second. Accordingly, the control data displays differently. Each clinical isolate is displayed in a separate graph for clarity in presentation and ease of comparisons to the prototype strain SC5314. Data are the mean values and SEM from 2 independent experiments involving 4 different colonies of each Candida strain (each colony was treated as a biological replicate) and at least 2000 macrophages surveyed for each strain, per experiment. The data for strains P87, L26, P78048, P57055, P78042 and P75010 shown here is the same as the data shown in Fig 1C. Here we show it again to have a complete set of C. albicans clinical strains for comparison. (TIF) [file ppat.1008695.s003.tif]

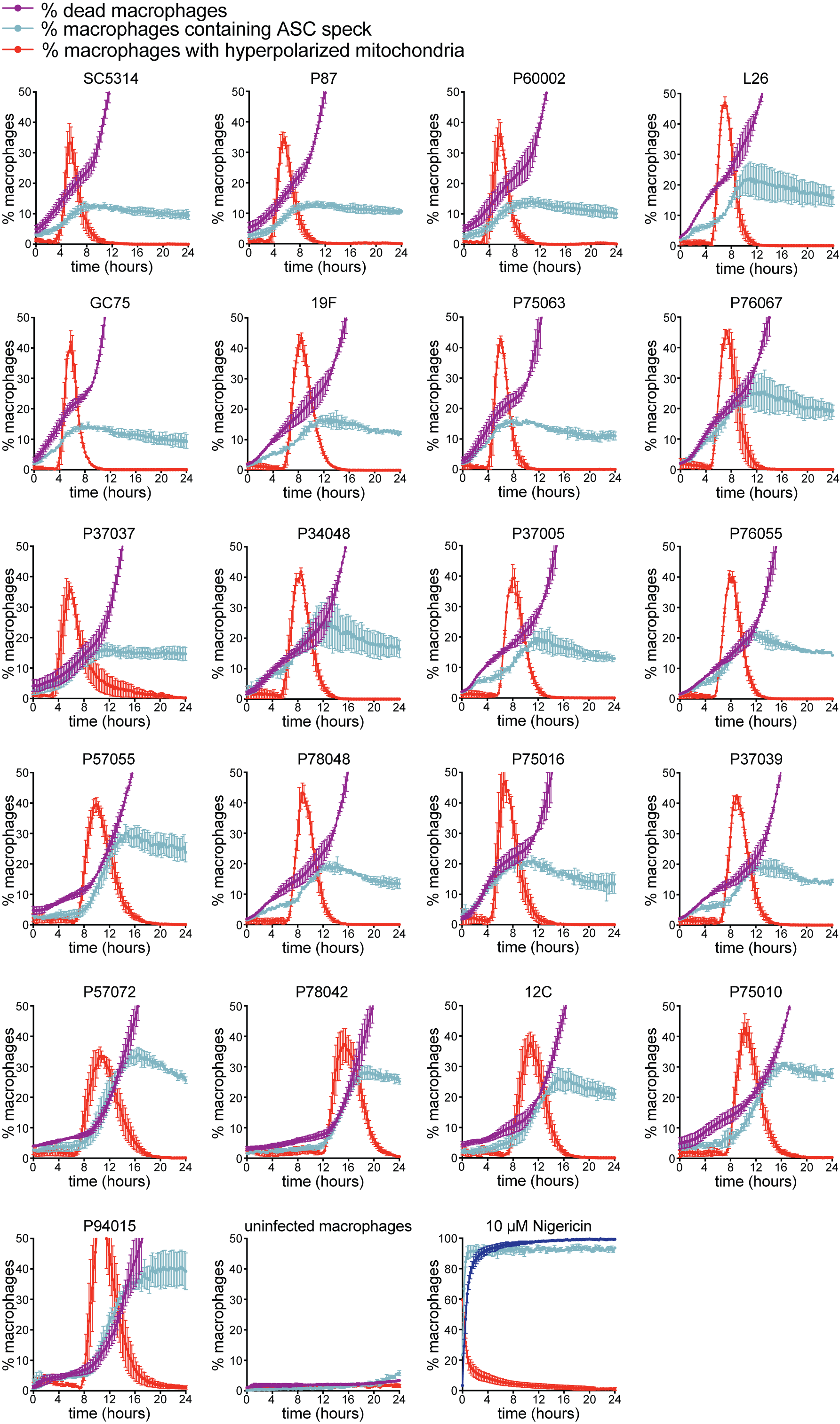

Supplement: S4 Fig — Macrophages were infected with the indicated C. albicans strains at 6:1 MOI or treated with nigericin (10 μM). Displayed in each graph are % dead macrophages, % macrophages containing an ASC speck, and % macrophages with hyperpolarized mitochondria as quantified in live cell imaging experiments. Data are the mean values and SEM from 3 independent experiments. The data for SC5314 and P78042 is the same as shown in Fig 3. Here we show it again to have a complete set of clinical isolates for comparison. The y-axis is set to 50% maximum to show clearly the ASC speck formation events, but macrophage cell death continued accumulating and eventually reached 100% for all C. albicans strains. We note that with the ASC-mCerulean expressing immortalised BMDMs the kinetics of macrophage cell death in response to C. albicans clinical strains was generally faster compared to experiments with primary BMDMs. Nevertheless, as in primary BMDMs, in the ASC-mCerulean immortalised cells the C. albicans strains that form robust hyphae triggered macrophage cell death more rapidly than those with less robust hyphal formation, meaning that the relationship between morphogenesis and macrophage cell death was preserved. (TIF) [file ppat.1008695.s004.tif]

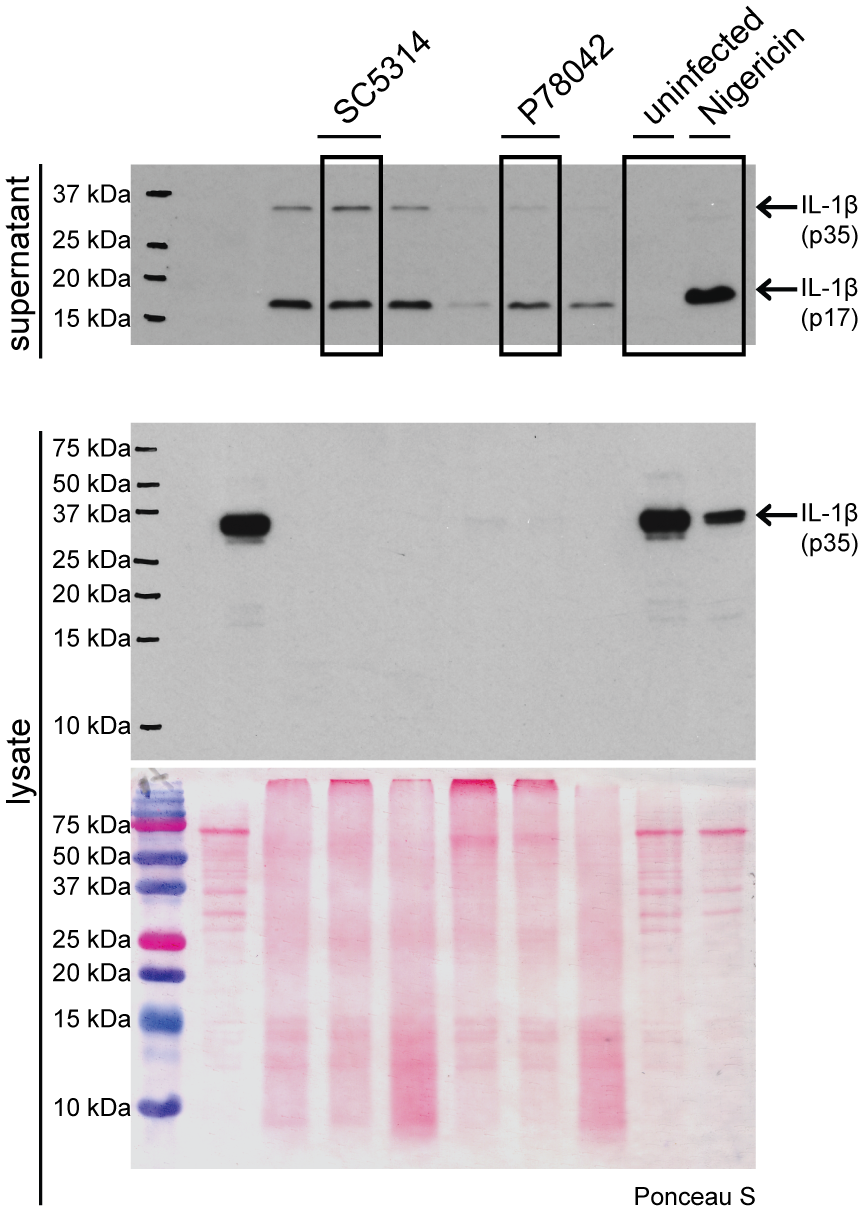

Supplement: S5 Fig — Murine bone marrow-derived macrophages were primed with LPS (50 ng/ml) for 3 h, followed by challenge with Candida strains SC5314 or P78042 (MOI 6:1), nigericin (10 μM), or uninfected. At 12 h post-challenge, supernatants and lysates were collected and analysed by immunoblot for IL-1β. Bottom image contains Ponceau S staining of total protein from lysates. Displayed in the boxes are the lanes that were spliced together to construct Fig 3E. (TIF) [file ppat.1008695.s005.tif]

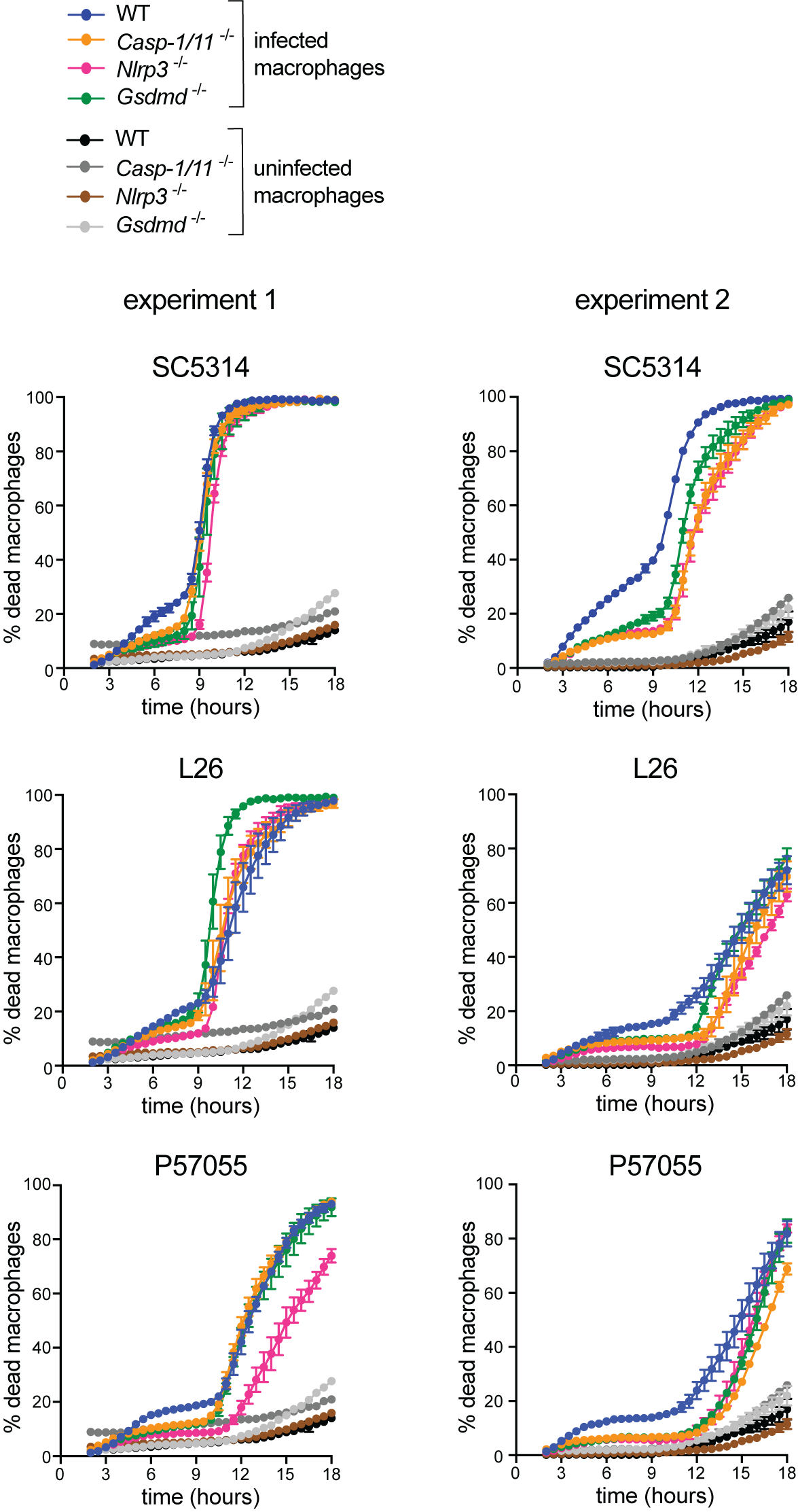

Supplement: S6 Fig — Wild type, Casp1/11-/-, Nlrp3-/- and Gsdmd-/- murine bone marrow-derived macrophages were primed with LPS (50 ng/ml) for 3 h followed by infection with C. albicans strains SC5314, L26 and P57055 at 6:1 MOI. Experiment 1 and 2 were performed on two different days, using the same preparations of murine BMDMs from each inflammasome mutant (one mouse per strain) and independent cell cultures of each C. albicans clinical isolate. In each of the experiments, all data were collected together, but each C. albicans isolate is displayed in a separate graph for clarity. The uninfected controls are the same in the three graphs from the same experiment. Shown are the average and SEM of 3 technical repeats for Candida infections and two technical repeats for uninfected controls. At least 3000 macrophages were counted for each of the samples. (TIF) [file ppat.1008695.s006.tif]

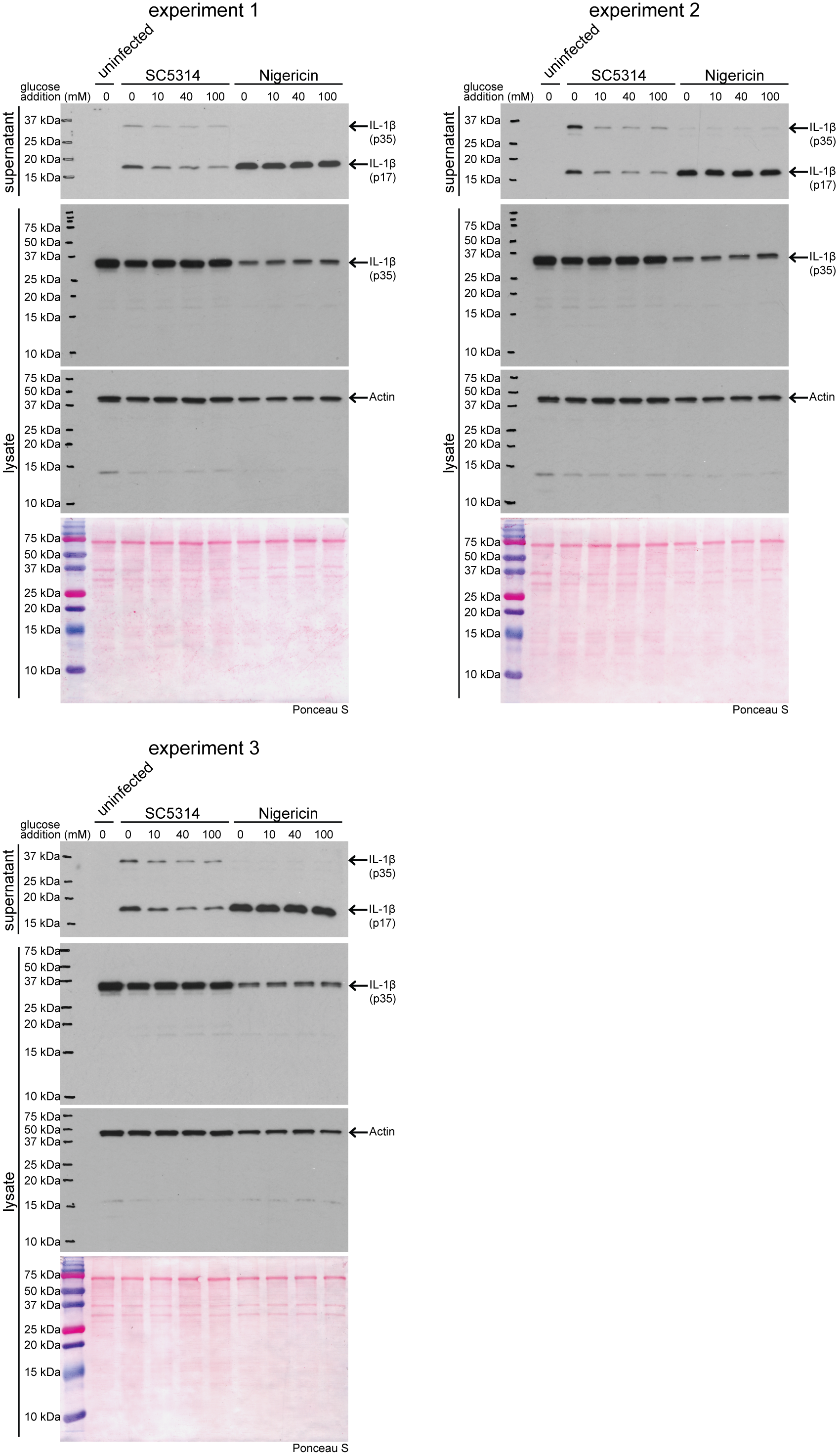

Supplement: S7 Fig — Murine bone marrow-derived macrophages (harvested independently from three different mice) were primed with LPS (50 ng/ml) for 3 h, followed by treatment with C. albicans hyphal strain SC5314 (MOI 6:1), nigericin as positive control (10 μM), or uninfected as negative control. The starting tissue culture media (~0.65 mM glucose) contained 0, 10, 40, or 100 mM added glucose. At 3 h post-infection, supernatants and lysates were collected and analysed by immunoblot. For each experiment, bottom image contains Ponceau S staining of total protein from lysates. (TIF) [file ppat.1008695.s007.tif]

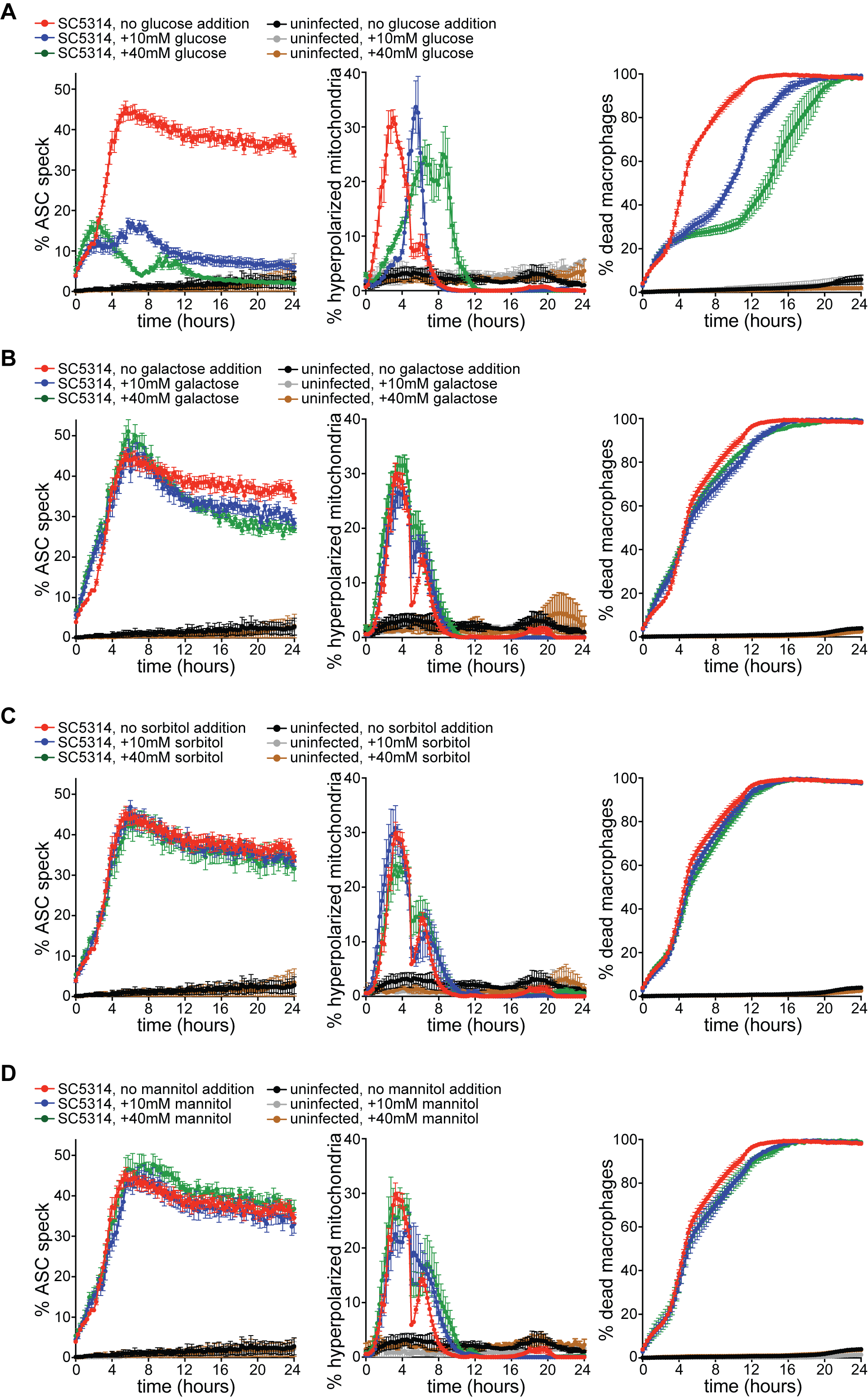

Supplement: S8 Fig — The conditions in panels A-D were all assayed in the same live cell imaging experiments; therefore, the data for control conditions (with no carbon source addition) are the same between different panels. For clarity in presentation, the data are displayed in separate graphs. (A) ASC-mCerulean-expressing immortalized macrophages following infection with Candida hyphal strain SC5314 (MOI 6:1) with the tissue culture media (~0.65 mM glucose) containing 0, 10, or 40 mM added glucose. Shown are % macrophages containing an ASC speck, % macrophages with hyperpolarized mitochondria, and % dead macrophages, as quantified in live cell imaging experiments. Data are the mean values and SEM from 2 independent experiments involving at least 9000 macrophages surveyed for each condition, per experiment. (B) Same as panel A, but with the tissue culture media containing 0, 10, or 40 mM added galactose (note that the “no galactose addition” controls are the same data as the “no glucose addition” controls in panel A). (C) Same as panel A, but with the tissue culture media containing 0, 10, or 40 mM added sorbitol (note that the “no sorbitol addition” controls are the same data as the “no glucose addition” controls in panel A). (D) Same as panel A, but with the tissue culture media containing 0, 10, or 40 mM added mannitol (note that the “no mannitol addition” controls are the same data as the “no glucose addition” controls in panel A). (TIF) [file ppat.1008695.s008.tif]
