# Supplementary material for: Metabolic competition between host and pathogen dictates inflammasome responses to fungal infection
Source: PLoS Pathog. 2020 Aug 4;16(8):e1008695. doi: 10.1371/journal.ppat.1008695 (PMC7433900; doi:10.1371/journal.ppat.1008695)
Supplement: S1 Table — (DOCX) [file ppat.1008695.s010.docx]

**Table S1. Strains used in this study.**

| Strain # | Name | Description | Genotype | Parent | Source |
| --- | --- | --- | --- | --- | --- |
| YCAT229 | SC5314 | Clinical isolate, from patient with “*generalised Candida infection*” (Odds et al Genome Biology 2004, 5: 230 | Clade 1 |  | Traven lab |
| YCAT793 | GC75 | Clinical isolate, H (or) | Clade 4 |  | BEI Resources, NIAID, NIH: *Candida albicans*, Strain GC75, NR-29452. |
| YCAT794 | P75016 | Clinical isolate, BSI | Clade 4 |  | BEI Resources, NIAID, NIH: *Candida albicans*, Strain P75016, NR-29438. |
| YCAT795 | P75063 | Clinical isolate, BSI | Clade 4 |  | BEI Resources, NIAID, NIH: *Candida albicans*, Strain P75063, NR-29440 |
| YCAT796 | P87 | Clinical isolate, HIV+ (or) | Clade 4 |  | BEI Resources, NIAID, NIH: *Candida albicans*, Strain P87, NR-29453. |
| YCAT797 | P60002 | Clinical isolate, BSI | Clade 8 |  | BEI Resources, NIAID, NIH: *Candida albicans*, Strain P60002, NR-29448. |
| YCAT798 | P94015 | Clinical isolate, BSI | Clade 6 |  | BEI Resources, NIAID, NIH: *Candida albicans*, Strain P94015, NR-29446. |
| YCAT799 | P34048 | Clinical isolate, BSI | Clade 3 |  | BEI Resources, NIAID, NIH: *Candida albicans*, Strain P34048, NR-29436 |
| YCAT800 | P78042 | Clinical isolate, BSI | Clade 3 |  | BEI Resources, NIAID, NIH: *Candida albicans*, Strain P78042, NR-29443. |
| YCAT801 | P57055 | Clinical isolate, BSI | Clade 3 |  | BEI Resources, NIAID, NIH: *Candida albicans*, Strain P57055, NR-29439. |
| YCAT802 | P57072 | Clinical isolate, BSI | Clade 2 |  | BEI Resources, NIAID, NIH: *Candida albicans*, Strain P57072, NR-29435. |
| YCAT803 | P76055 | Clinical isolate, BSI | Clade 2 |  | BEI Resources, NIAID, NIH: *Candida albicans*, Strain P76055, NR-29441 |
| YCAT804 | P76067 | Clinical isolate, BSI | Clade 2 |  | BEI Resources, NIAID, NIH: *Candida albicans*, Strain P76067, NR-29442. |
| YCAT805 | P75010 | Clinical isolate, BSI | Clade 11 |  | BEI Resources, NIAID, NIH: *Candida albicans*, Strain P75010, NR-29437 |
| YCAT806 | 19F | Clinical isolate, VP(v) | Clade 1 |  | BEI Resources, NIAID, NIH: *Candida albicans*, Strain 19F, NR-29449. |
| YCAT807 | L26 | Clinical isolate, VP(v) | Clade 1 |  | BEI Resources, NIAID, NIH: *Candida albicans*, Strain L26, NR-29445. |
| YCAT808 | P37039 | Clinical isolate, H(or) | Clade 1 |  | BEI Resources, NIAID, NIH: *Candida albicans*, Strain P37039, NR-29451. |
| YCAT809 | 12C | Clinical isolate, VP(oral) | Clade 1 |  | BEI Resources, NIAID, NIH: *Candida albicans*, Strain 12C, NR-29444 |
| YCAT810 | P37005 | Clinical isolate, H(or) | Clade 1 |  | BEI Resources, NIAID, NIH: *Candida albicans*, Strain P37005, NR-29447. |
| YCAT811 | P37037 | Clinical isolate, H(or) | Clade 1 |  | BEI Resources, NIAID, NIH: *Candida albicans*, Strain P37037, NR-29450. |
| YCAT812 | P78048 | Clinical isolate, BSI | Clade 1 |  | BEI Resources, NIAID, NIH: *Candida albicans*, Strain P78048, NR-29434. |
| YCAT834 | CAS9 | *tye7∆/∆ gal4∆/∆* | *tye7∆∆gal4∆∆*  *tye7∆::FRT/tye7∆::FRT*  *gal4∆::ARG4/gal4∆::HIS1*  *RPS10/rps10::URA3* | BWP17 | Askew et al *PLoS Pathogens* 2009, e1000612, PMID 19816560 |
| YCAT835 | CAS10 | *tye7∆/∆ gal4∆/∆*  *+TYE7+GAL4* | *tye7∆∆gal4∆∆*+*TYE7*+*GAL4*  *tye7∆::FRT/TYE7::FRT*  *gal4∆::ARG4/GAL4::FRT*  *HIS1/his1::hisG, RPS10/rps10::URA3* | BWP17 | Askew et al *PLoS Pathogens* 2009, e1000612, PMID 19816560 |

Abbreviations in table: BSI, bloodstream isolate; VP, vaginitis patient; v, vaginal sample;

or, oral sample; HIV+, HIV-positive; H, healthy individual
